# Exploring the potential of OMOP common data model for process mining in healthcare

**DOI:** 10.1371/journal.pone.0279641

**Published:** 2023-01-03

**Authors:** Kangah Park, Minsu Cho, Minseok Song, Sooyoung Yoo, Hyunyoung Baek, Seok Kim, Kidong Kim

**Affiliations:** 1 Department of Industrial and Management Engineering, Pohang University of Science and Technology (POSTECH), Pohang, South Korea; 2 School of Information Convergence, Kwangwoon University, Seoul, South Korea; 3 Healthcare ICT Research Center, Office of eHealth Research and Businesses, Seoul National University Bundang Hospital, Seongnam, South Korea; 4 Department of Obstetrics and Gynecology, Seoul National University Bundang Hospital, Seongnam, South Korea; University of Pannonia: Pannon Egyetem, HUNGARY

## Abstract

**Background and objective:**

Recently, Electronic Health Records (EHR) are increasingly being converted to Common Data Models (CDMs), a database schema designed to provide standardized vocabularies to facilitate collaborative observational research. To date, however, rare attempts exist to leverage CDM data for healthcare process mining, a technique to derive process-related knowledge (e.g., process model) from event logs. This paper presents a method to extract, construct, and analyze event logs from the Observational Medical Outcomes Partnership (OMOP) CDM for process mining and demonstrates CDM-based healthcare process mining with several real-life study cases while answering frequently posed questions in process mining, in the CDM environment.

**Methods:**

We propose a method to extract, construct, and analyze event logs from the OMOP CDM for process types including inpatient, outpatient, emergency room processes, and patient journey. Using the proposed method, we extract the retrospective data of several surgical procedure cases (i.e., Total Laparoscopic Hysterectomy (TLH), Total Hip Replacement (THR), Coronary Bypass (CB), Transcatheter Aortic Valve Implantation (TAVI), Pancreaticoduodenectomy (PD)) from the CDM of a Korean tertiary hospital. Patient data are extracted for each of the operations and analyzed using several process mining techniques.

**Results:**

Using process mining, the clinical pathways, outpatient process models, emergency room process models, and patient journeys are demonstrated using the extracted logs. The result shows CDM’s usability as a novel and valuable data source for healthcare process analysis, yet with a few considerations. We found that CDM should be complemented by different internal and external data sources to address the administrative and operational aspects of healthcare processes, particularly for outpatient and ER process analyses.

**Conclusion:**

To the best of our knowledge, we are the first to exploit CDM for healthcare process mining. Specifically, we provide a step-by-step guidance by demonstrating process analysis from locating relevant CDM tables to visualizing results using process mining tools. The proposed method can be widely applicable across different institutions. This work can contribute to bringing a process mining perspective to the existing CDM users in the changing Hospital Information Systems (HIS) environment and also to facilitating CDM-based studies in the process mining research community.

## Introduction

Over the past decade, the amount of healthcare data has been on a significant increase. With the abundance of data, medical institutions seek to improve both clinical outcomes and operational efficiency. To this end, hospital processes are analyzed to obtain process-related knowledge. Hospital processes can be classified into several categories. Some specify two types of hospital processes: medical treatment processes and organizational processes [1–3], while others divide healthcare processes into non-elective care and elective care [4]. Multiple process types are discussed in analyzing hospital processes: inpatient, outpatient, emergency room processes, and patient journey. The former three pertain to the purpose of each visit: to have a surgical operation, to consult with a physician and receive proper procedure or medication, or to receive immediate care. Patient journey refers to a long-term flow of events from the patient’s perspective, encompassing a course of patient encounters in the care site during a predefined period.

Process mining is a research discipline frequently used to analyze these hospital processes. The primary purpose of process mining is to derive process-related knowledge from event logs, a dataset that contains information on event occurrences at different times. Of the several application areas of process mining, healthcare process analysis is one of the popular topics gaining increasing attention. In the healthcare domain, events such as treatments and examinations during a single visit are delineated by order of occurrence to be illustrated as a flow to form a process model. This fundamental task is referred to as process discovery or control-flow analysis in process mining.

Several process mining contributions have dealt with various challenges in medical domains [5]. Thus far, a large part of clinical data used for healthcare process mining has been sourced from hospitals’ EHR systems, which are often highly heterogenous across organizations. As a result, event logs have been formulated in numerous different ways according to the structure of the EHR data in use in the absence of the common data structure and vocabularies. Likewise, disseminating and reproducing analytical results have also been challenging due to the disparate data structures and representations in different sites [6].

Common Data Models (CDMs) are a database schema designed to address this particular issue. It is defined as “a set of uniform data standards that regulate the format and content of observational data, support observational data from different sources, and form a standardized data structure through data Extraction-Transformation-Loading (ETL) [7].” Of the several CDMs, the Observational Medical Outcomes Partnership (OMOP) CDM is one of the widely accepted models, while the conversion of clinical observational data from the EHR to CDM is in progress. Since the Observational Health Data Sciences and Informatics (OHDSI)’s foundation in 2014, literature on OMOP CDM (referred to as CDM hereafter for brevity) has steadily increased. The number of studies on CDM in 2020 has doubled from the previous year’s [8], and more than 330 databases in over 30 countries have been converted to CDM as of 2020 [9]. A growing number of institutions around the globe are adopting CDM as a standard healthcare database.

Recent works that employ CDM are centered on the ETL stage and the applications after the ETL. The ETL stage deals with the database conversion from the EHR to CDM. Research attempts have been made by mapping vocabularies with CDM components following an ETL framework [10], addressing issues during the conversion process [11], or evaluating the feasibility of implementation in various aspects (e.g., data quality) [12–14]. Among the few papers that discuss CDM application after ETL, Zhang et al. [7] extract data from a Chinese hospital to examine treatment pathways of three chronic diseases. They present treatment pathways in sunburst plots using an OHDSI visualization tool. Similarly, Byun et al. [15] analyze treatment patterns of anti-dementia medications using CDM-extracted data. These studies employ open-source CDM applications provided by OHDSI for data analysis and visualization (e.g., the Sankey diagram). Boudis et al. [16] and Lamer et al. [17] also discover and visualize patient movements in Sankey diagrams for multiple surgical procedures. Although these studies leverage CDM in some ways, none of them delves into process analysis, lacking process mining perspectives. As for studies that exploit CDM on the process mining side, only a limited number of studies are found. Cho [18] presents a query to extract event logs from CDM and Choi [19] suggests a process to extract and analyze clinical pathways (CPs) in a CDM environment.

Despite the rare attempts to leverage CDM for process mining purposes, it will become more crucial to be able to adopt CDM as the transition of healthcare data sources continues [10–13, 20]. Consequently, the need for a universal method for event log preparation and analysis in the midst of CDM’s growing presence will increase as well. This paper aims to address this research need by suggesting steps for CDM-based healthcare process mining by demonstrating event log extraction with a customizable query and reproducible process analysis with real-world CDM data.

The migration from the EHR system to CDM has clear benefits in coping with process mining challenges as well. Three of the ten challenges outlined in [21] are particularly relevant to the transition to CDM (i.e., C7, C8, C9). First, CDM has strengths in ensuring patient privacy (i.e., C7: Take Care of Privacy and Security) since de-identification strategies are designed and applied throughout the ETL process, as demonstrated in [22, 23].

Second, CDM helps researchers view the process from the perspective of patients (i.e., C8: Look at the Process through the Patient’s Eyes). Having a patient’s perspective means to consider the clinical history of a patient when providing care, as opposed to the manager’s perspective that considers hospital’s operational efficiency (e.g., waiting time, LOS). Studying a process from the patient’s viewpoint can encourage physicians to take the patient journey into consideration when making clinical decisions [21]. As patient-level prediction is now frequently conducted in CDM-based research as an increased volume of clinical data become available in CDM [24, 25], in this paper we also intend to bring patients’ perspective by discussing patient journey discovery in a CDM environment.

**Fig 1:**
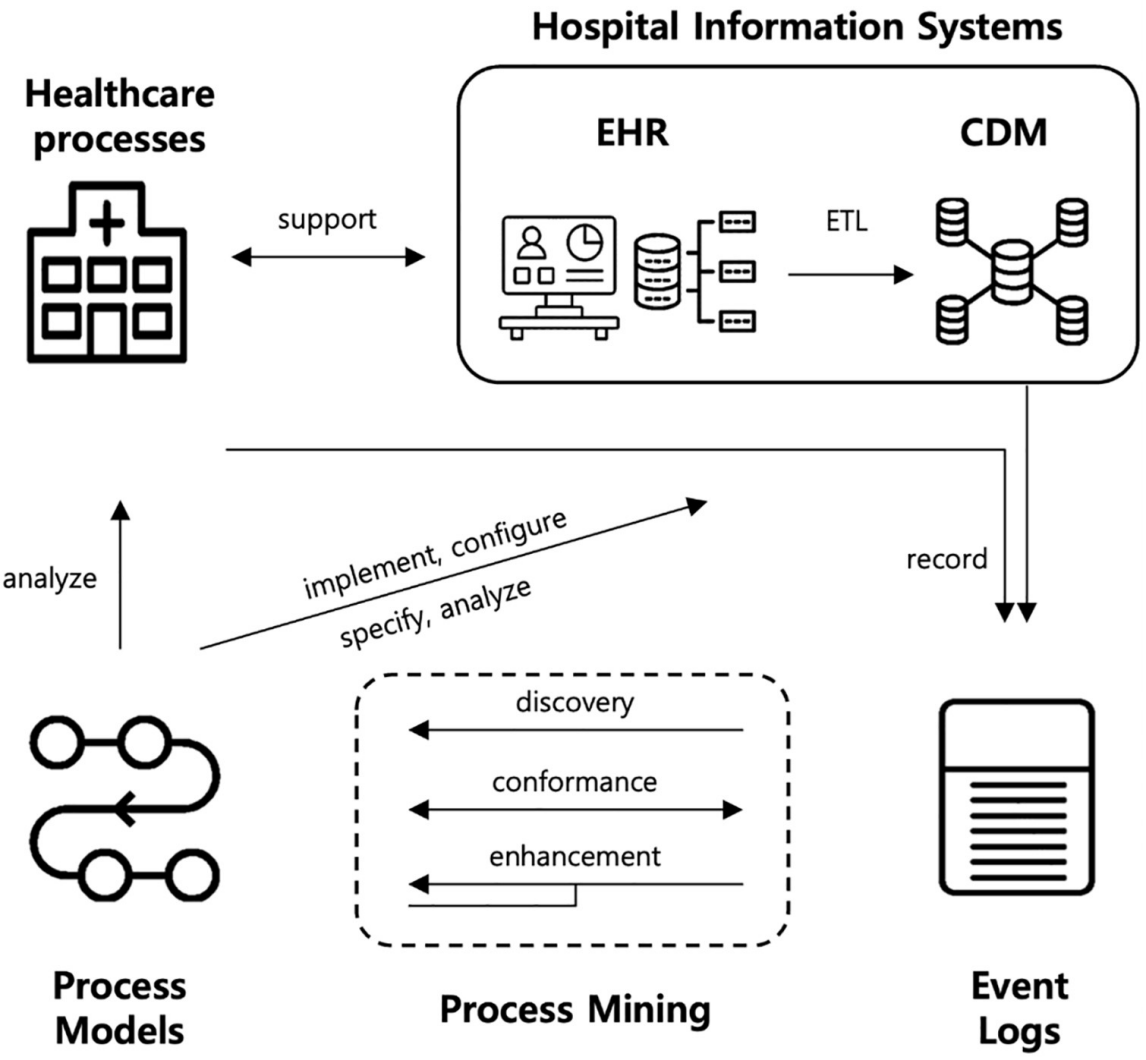
Process mining in the changing HIS environment (adapted based on [21, 26]).

Lastly, this paper aims to address challenge ‘C9: complement Hospital Information Systems (HISs) with the process perspective’, which pertains to the source of healthcare data. The HISs including traditional EHR and more integrated systems (e.g., CDM) can benefit from being supplemented with the process perspective [21]. As an attempt to complement CDM with the process mining perspective, we identify relevant data elements in CDM and the additional data requirements to be incorporated into CDM to open the door for process mining. The changing HIS environment and the interplay between healthcare processes, process mining, process models, event logs are illustrated in Fig 1.

We define four process types in this paper: inpatient, outpatient, emergency room (ER) process, and patient journey (PJ). As mentioned earlier, we use ‘patient journey’ to refer to a series of events that occurred to a patient or patient cohort during several years, distinguishing it from ‘episodes.’ Episodes in the healthcare setting are of several types: episodes of illness (patient’s perspective), episodes of care (provider’s perspective), episodes of disease, and healthcare maintenance episodes [27]. Although ‘episode of care’ refers to a similar concept to PJ, it does not necessarily consider the visit types (i.e., inpatient, outpatient, ER visit), which gives us the ground for adopting the term ‘patient journey’ instead of an ‘episode.’

For each process type, EHR-based process mining studies are available. First, inpatient process analysis covers a wide range of topics related to clinical pathways (CP). CP analysis includes process discovery [17], CP variance management [28, 29], temporal pattern mining (e.g., Length of Stay) [30, 31], personalized CP generation [32, 33], among others. While some papers on CP cover the entire process ranging from pre-operational to post-operational treatment activities [34], others investigate surgical processes per se by analyzing activities undertaken inside the operation room [35]. Typically, activities in an inpatient process include clinical orders related to procedures and medications and measurement results from vital checks or lab tests.

Second, outpatient processes are frequently discussed using operational-level activities ranging from registration, measurement, consultation, prescription issuance, consultation reservation, to payment [36–39], including clinical and non-clinical activities. Besides process discovery, the performance perspective is commonly discussed when analyzing outpatient visit logs. In existing studies, wait times are frequently analyzed [40, 41]. For conformance checking, discovered process models are evaluated by the matching rate [42] or by an adherence measurement algorithm [43], which measures the derived model’s degree of conformance to the reference model [37, 40, 42].

Third, ER processes are frequently analyzed from the resource perspective. Existing studies examine collaboration patterns between different healthcare professionals (e.g., nurse, physician, medical assistant) using network analysis [44] as well as the interplay of departments using a role interaction model [45]. Since collaboration and process efficiency are imperative in ER, optimization of an ER layout [46] or a development of process performance indicators for ER [47] is also performed.

Lastly, PJ analysis provides an enlarged picture. For example, process models are discovered and visualized using the visit data of chronic kidney disease patients who had developed acute kidney injury between 2009 and 2013 [48]. In this study, patient visits are categorized based on their purposes: education, office, and hospital. In another study, patients’ conditions before and after clinical interventions are examined to determine the effectiveness of treatments [49].

In the following sections, we illustrate steps to extract event logs from the CDM of a tertiary hospital by using queries for different process types. The extracted logs are then used for process analysis to demonstrate CDM-based healthcare process mining. In doing so, we focus on the data extraction and analysis stages after the ETL (i.e., data conversion) instead of data conversion, assuming that the quality of the conversion is guaranteed.

## Methods

### Locate relevant CDM tables for event log formulation

**Fig 2:**
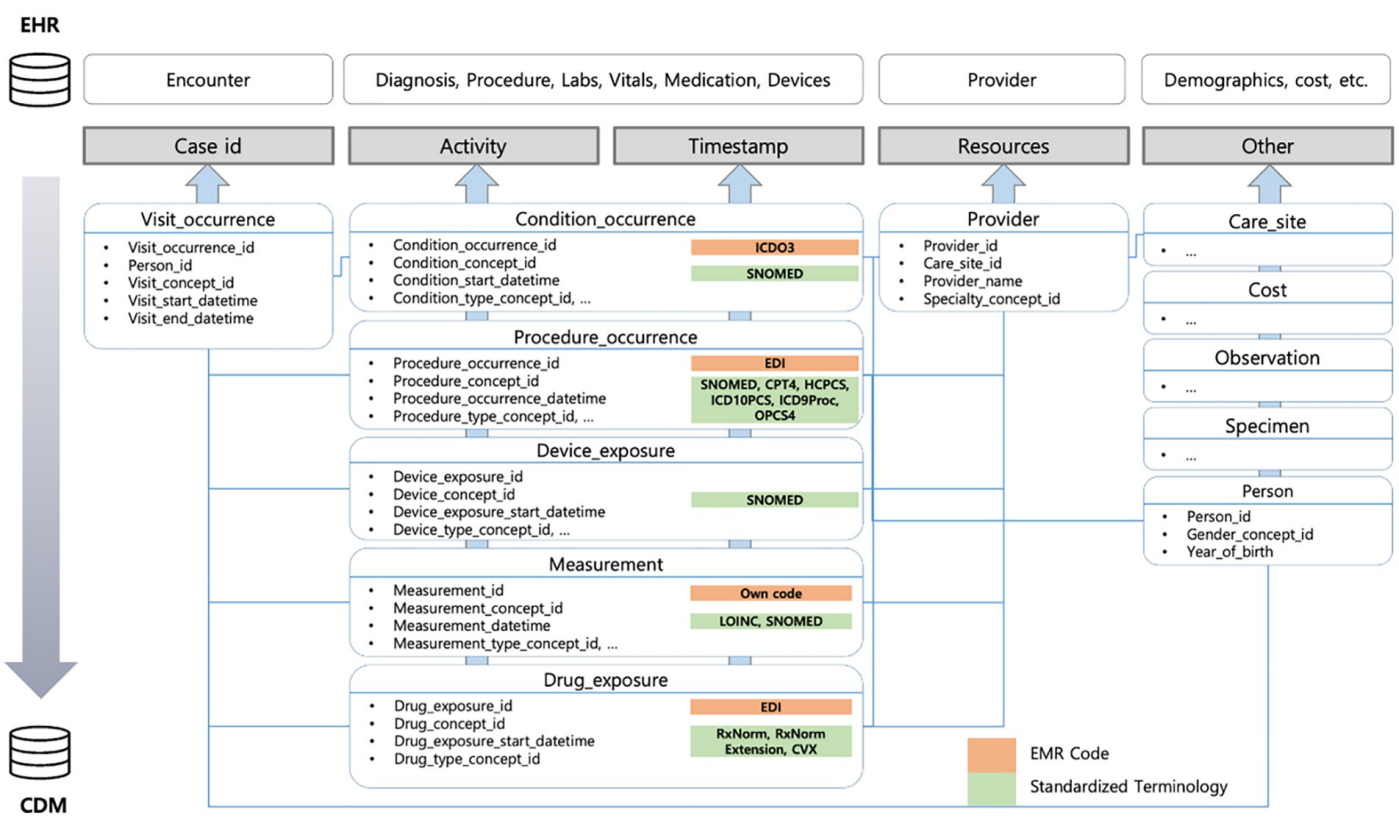
Relevant CDM tables for event log construction. Code names are sourced from [50].

The relevant CDM tables (version 5.3 or later) required to construct event logs are presented in Fig 2. Event logs are comprised of case identifiers (case IDs), activities, timestamps, and other attributes such as resources and care sites. Case IDs are generated for each patient encounter, while activities and timestamps are logged when clinical events such as diagnosis, procedure, laboratory test, vital check, and prescription occur. In CDM, encounters are found in *visit_occurrence* table, which has person_id, provider_id, and care_site_id as foreign keys so that information on each visit can readily be derived.

Activities and timestamps are found within five CDM tables: *condition_occurrence*, *procedure_occurrence*, *device_exposure*, *measurement*, and *drug_exposure*. These tables contain information on clinical interventions undertaken during each patient visit. *Condition_occurrence* contains information on diagnosis or chief complaints. *Procedure_occurrence* and *Device_exposure* describe surgical operations and procedures taken and use of medical devices during each encounter, respectively, while *Measurement* table contains lab results and vital signs. *Drug_exposure* is concerned with prescribed or administered medications, which are mapped in CDM using standardized terminology such as Rx-Norm. Information on resources can be found in *Provider* table, while other attributes can be sourced from *Care_site*, *Cost*, *Observation*, or *Person* [51].

### Define elements for process mining

To construct event logs within a CDM environment, we should specify required and optional elements and determine the ETL process based on the event log specification. We specify required and optional elements by using five items: process type, period, scope, field, and feature. **Process type** refers to the visit types defined above. For the former three, concept IDs (i.e., *visit_concept_id* in *visit_occurrence* table) should be given as 9201 (Inpatient visit), 9202 (Outpatient visit), and 9203 (Emergency Room visit), respectively. In some cases, concept ID 262 can be used for Emergency Room and Inpatient visits. **Period** indicates a span of time during which the events in concern had occurred. **Scope** determines which clinical activities should be included in or excluded from event logs. **Field** identifies elements to be included in event logs. Namely, case IDs, event IDs, case attributes, and event attributes fall into the field category. In general, case IDs, event IDs, and three event attributes (i.e., activities, timestamps, and originators) are the minimum requirements to form event logs. **Feature** refers to optional elements used to enrich the analysis by providing additional information. For example, the location where clinical activities had occurred can be used at different levels of granularity using *care_site* table for a particular purpose.

### Generate a query to extract event logs

This section describes ways in which relevant attributes are extracted from CDM and formulated as event logs. To this end, we develop a query in Structured Query Language (SQL) in S1 Table. The first part defines columns to use in the selected tables. In general, the case ID should be ‘visit_occurrence_id’, taken from visit_occurrence table. Alternatively, we can also consider using ‘person_id’ in the person table as the case ID for the patient journey. Event IDs are obtained from the tables found in the scope part (e.g., ‘procedure_occurrence_id’ in procedure_occurrence table). Required event attributes (i.e., activity, timestamp, and originator) are derived from ‘procedure_concept_id’, ‘procedure_datetime’, and ‘provider_id’ under procedure_occurrence table. The second part describes ways in which tables are selected and joined to constitute event logs. In this process, the table that represents the case (i.e., visit) is constructed by combining visit_occurrence, person, and provider tables.

### Customize the query for each process type

#### Inpatient process

Inpatient visits are coded as 9201 for ‘visit_concept_id.’ Clinical codes that indicate types of treatment and medication serve as activities in inpatient visit logs. Thus, a code that corresponds to a specific operation from ‘procedure_concept_id’ in *procedure_occurrence* table should be selected. Alternatively, a code for a primary diagnosis could be selected from ‘condition_concept_id’ within *condition_occurrence* table. *Scope* should be defined to cover *measurements*, *drug_exposure*, *procedure_occurrence* in CDM since CP involves all of these clinical activities.

As mentioned earlier, patients’ LOS during hospitalization is a research topic from the time perspective. Hospitals are desired to have the LOS under control since it can serve as a key performance indicator for patient safety and operational efficiency. The LOS for each patient can be calculated by the patient’s ‘visit_start_datetime’ and ‘visit_end_datetime’, both of which are found in *visit_occurrence* table. The LOS can be added as one of the case attributes. Additional case attributes such as patient’s demographics, providers in charge, and care site can also be added for a detailed analysis. Table 1 shows example event log for inpatient process analysis. As shown in the table, activities are in numerical code, which can be found in the CDM vocabulary and matched to obtain corresponding descriptions. For this case, we provide explicit activity names along with the numerical code for the activity column.

**Table 1:** An example of inpatient visit event logs.

Case ID	Event ID	Event attributes			Case attributes			
		Activity	Timestamp	Originator	LOS	Sex	Ages	…
**C10001**	E10001	1154186 (fentanyl 0.1 MG)	2021-08-01 09:03:00	O10001	3	8532 (Female)	40	…
	E10002	19086213 (midazolam 5 MG/ML)	2021-08-01 10:01:00	O10002	3	8532 (Female)	40	…
	E10003	3012608 (segmented neutrophils/100 leukocytes in blood by automated count)	2021-08-01 12:30:00	O10003	3	8532 (Female)	40	…
	E10004	3013869 (basophils/100 leukocytes in blood by automated count)	2021-08-01 12:35:00	O10004	3	8532 (Female)	40	…
**C10002**	E10005	1154186 (fentanyl 0.1 MG)	2021-08-02 10:01:00	O10005	4	8507 (Male)	50	…
**…**	…	…	…	…	…	…	…	…

#### Outpatient process

The ‘visit_concept_id’ for an outpatient visit is 9202. Contrary to inpatient process, an outpatient process typically lasts a few hours and is expected to provide an operational point of view. In general, the activities in an outpatient process include registration, scheduling for consultation, consultation, tests, prescription issuance, scheduling for registration, and payments. However, it is found that not all CDMs provide administrative events at a detailed level. Table 2 shows an example of activities that can be acquired from CDM. The activities are at a coarser level than those extracted from the EHR, although the level of abstraction of data from administrative systems are already high in many cases [52].

**Table 2:** Available outpatient activities derived from CDM.

Table	Coordinated outpatient activity
**Condition_occurrence**	Consultation
**Visit_occurrence**	
**Measurements**	Physical examination & Lab test
**Drug_exposure**	Drug
**Procedure_occurrence**	Surgery & Radiology test
**Cost**	Payment

To construct event logs for outpatient visits, the query in S1 Table should be modified accordingly. It should be noted that we may obtain a misleading activity instead of a clinical code by the command in line 4. For example, if the events had been extracted from *visit_occurrence* table, running the command in line 4 would return ’Consultation’ as activity. If necessary, information on involving departments can also serve as event attributes in analyzing an outpatient process. Table 3 shows an example event log that contains activity, timestamp, department, and originator as event attributes.

**Table 3:** An example of outpatient visit event logs.

Case ID	Event ID	Event attributes				Case attributes		
		Activity	Timestamp	Department	Originator	Sex	Ages	…
**C20001**	E20001	Physical examination	2021-08-01 10:01:00	Nursing Dept.	O20001	8532 (Female)	30	…
	E20002	Consultation	2021-08-01 10:10:00	Hematology oncology Dept.	O20002	8532 (Female)	30	…
	E20003	Lab test	2021-08-01 10:16:00	Internal medicine Dept.	O20003	8532 (Female)	30	…
	E20004	Drug	2021-08-01 10:25:00	Pharmaceutical care Dept.	O20004	8532 (Female)	30	…
**C20002**	E20005	Consultation	2021-08-01 10:04:00	Plastic surgery Dept.	O20005	8507 (Male)	50	…
…	…	…	…		…	…	…	…

#### Emergency room process

A different code, 9203, is used for ‘visit_concept_id’ to extract event logs for ER. ER processes have an explicit source of visits and a discharge destination. In CDM, ER visits and inpatient visits have ‘admitting_source_concept_id’ and ‘discharge_to_concept_id’ in *visit_occurrence* table. These can serve as the start and end activities of an ER process. The source of visits can vary: visit from home (‘8536’), patient transfer from hospital to hospital (‘44790567’, ‘8716’, ‘8717’), and transfer from outpatients (‘8756’). Table 4 provides an example event log for ER visits.

**Table 4:** An example of ER visit event logs.

Case ID	Event ID	Event attributes				Case attributes		
		Activity	Timestamp	Department	Originator	Sex	Ages	…
**C30001**	E30001	8536 (Visit from home)	2021-08-01 19:04:00	ER Dept.	O30001	8532 (Female)	10	…
	…	…	…	…	…	…	…	…
	E30004	Cooperative Care	2021-08-01 19:31:00	Pediatric Dept.	O30004	8532 (Female)	10	…
	…	…	…	…	…	…	…	…
	E30008	8536 (Discharge to home)	2021-08-01 20:04:00	ER Dept.	O30008	8532 (Female)	10	…
**C30002**	E30009	44790567 (Patient transfer from hospital to hospital)	2021-08-01 20:01:00	ER Dept.	O30009	8507 (Male)	50	…
…	…	…	…	…	…	…	…	…

#### Patient journey

The above processes combined altogether can provide a patient journey, a long-term care flow experienced by patients within a predefined period. A patient journey provides a patient-oriented view, taking patients’ visit history into consideration. Event logs for a patient journey can be obtained by selecting ‘person_id’ as the case ID and ‘visit_concept_id’ as a process type ID, both of which are found in *visit_occurrence* table. Table 5 shows an example event log for a patient journey. Activities for a patient journey can either be process types in a sequence (i.e., outpatient > outpatient > inpatient > emergency room), or be indexed activities annotated with specific events occurred during each visit. For example, with indexed activities, the process can be expressed as follows: outpatient1_drug > outpatient1_procedure >… > outpatient2_drug > inpatient1_drug > inpatient1_surgery > … > ER1_radiology > … > ER1_payment.

**Table 5:** An example log for patient journey.

Case ID	Event ID	Event attributes					Case attributes		
		Activity	Process type	Timestamp	Department	Originator	Sex	Age	…
**C40001**	E40001	Outpatient visit	9202	2021-08-01 09:04:00	OG	O30001	8532 (Female)	10	…
	E40002	Laboratory visit	32036	2021-08-01 10:04:00			8532 (Female)	10	
	E40003	Emergency room visit	9203	2021-09-11 12:04:00	ER	P20001	8532 (Female)	10	
	E40004	Inpatient visit	9201	2021-09-14 14:04:00	OG	G30003	8532 (Female)	10	
…	…	…		…	…	…	…	…	…

### Extract event logs and apply process mining techniques

In an effort to examine CDM’s ability to reproduce process mining analysis results as in EHR-driven research, we draw upon frequently posed questions in process mining for healthcare suggested by [52]. The questions are as follows: Research Question (RQ) 1: What are the most followed paths and exceptional paths?, RQ 2: Are there differences in care paths followed by different patient groups?, RQ 3: Do we comply with internal and external guidelines?, and RQ 4: Where are the bottlenecks in the process?

## Materials

We conduct empirical research using the CDM of a tertiary hospital in South Korea to demonstrate the utility of the proposed methods. To this end, the logs of Total Laparoscopic Hysterectomy (TLH) patients between 2003 and 2019 in the obstetrics and gynecology department are used for inpatient, outpatient, and patient journey. The TLH dataset contains visit cases of approximately 600 patients.

Particularly for inpatient process, we perform CDM-based CP discovery for four surgical procedures: total hip replacement (THR), coronary bypass (CB), transcatheter aortic valve implantation (TAVI), and pancreaticoduodenectomy (PD), which are the study cases used in [16, 17]. We also analyze an ER process using the CDM-based event log. Note that, as it is a retrospective observational study and the data source was de-identified, this study was approved based on waivers of informed consent or exemptions by the SNUBH Institutional Review Board (IRB No: X-2002–592–904).

### Inpatient process

The TLH dataset contains logs on medical interventions undertaken during the hospitalization of the selected patients. The number of events is 47,053 in total, where each patient received 83.7 clinical orders on average. The THR, CB, TAVI, and PD datasets contain 1,022, 458, 31, and 235 cases, each of which includes a single surgery occurrence and clinical orders between admission and discharge. We extract cases for the selected surgeries occurred between January 1, 2018, to December 31, 2021.

### Outpatient process

We select approximately 250 patients who received TLH in 2013 for the outpatient process. Events, cases, activities included in the raw dataset are 14,042, 2,998, and six, respectively. Since administrative events such as consultation or test registration and scheduling are not included in CDM, we use the names of selected tables: *condition_occurrence*, *observation*, *drug_exposure*, *procedure_occurrence*, *measurement*, and *cost*. Each of them refers to consultation, survey, medication, operation, treatment, or radiology test, laboratory test, and payment, respectively. For preprocessing, activities that only contain the date of occurrence without the exact times are given arbitrary timestamps to follow a predefined sequence: condition > drug > cost. We also set elapsed time between activities to five to 30 minutes depending on the number of repetitions.

### Emergency room process

We extract the event log for patient encounters at ER by following the proposed method. by following the proposed method. We initially extracted the log for the entire year of 2020, although we selected a month, December 2020, to present recognizable maps containing 2,652 visit cases of 2,503 patients with 82,018 events. The average LOS is 5.94 hours, with a standard deviation of 7.6 hours. As in the outpatient process, we use seven table names as activities due to the absence of detailed operational activities recorded in CDM.

### Patient journey

We select TLH patients hospitalized between January 2018 to December 2019 and filter visit cases that occurred from January 2017 to December 2020, to examine patient encounters before and after the operation. The dataset contains 7,003 visit cases of 683 patients who visited the hospital for four different purposes: inpatient, outpatient, ER, and Checkup. Since a process model reflecting all of the detailed events during each round of visits can be overly complicated, a visit-level process model is derived, including four visit types (i.e., Inpatient, Outpatient, ER, Checkup). For inpatient visits, we consider a single occurrence of hospitalization as one visit (i.e., In_1) for simplification.

## Results

In this section, we apply the proposed method to define and extract event logs from the real-life CDM and perform process analyses for each process type: inpatient, outpatient, ER, and PJ. Later in the discussion, we compared our inpatient case studies with the existing studies that were based on the same surgical operations. For the outpatient process, we compare the CDM-based process model with an EHR-based model and highlight the differences. Given the results, we answer the questions posed in [52].

### Inpatient process

**Fig 3:**
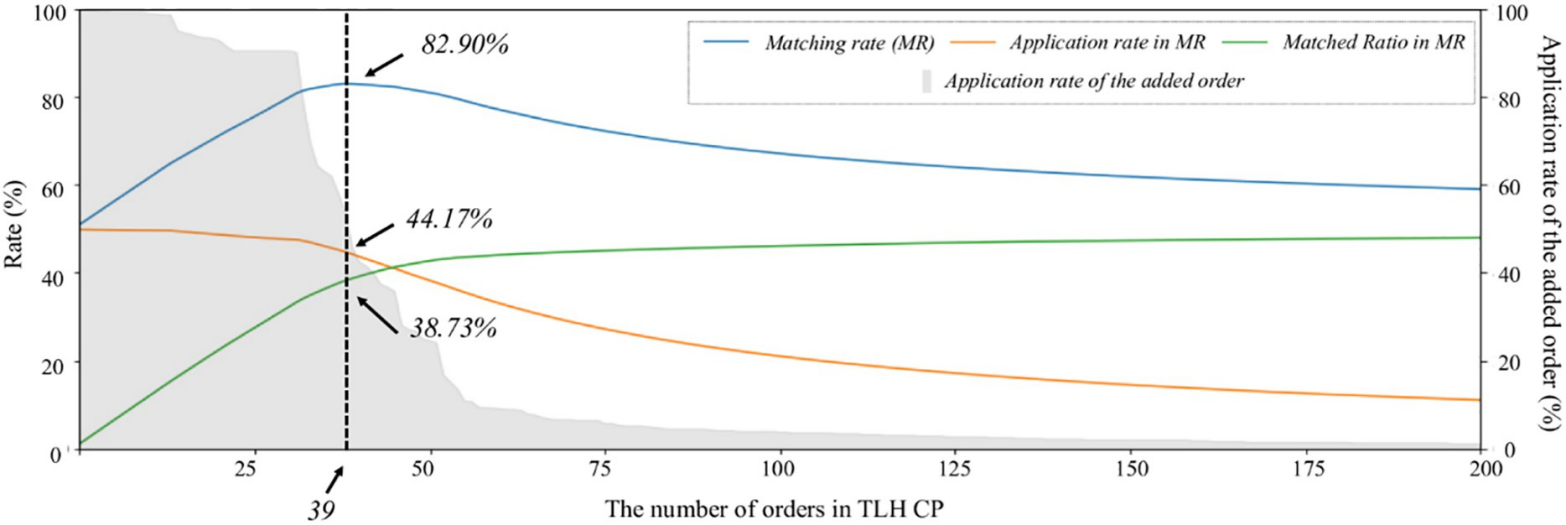
Finding the optimal number of clinical orders in TLH CP.

First, with the TLH dataset, we apply the matching rate-based CP mining algorithm proposed in [53] with the CDM-derived event logs. The algorithm aims to derive a series of clinical orders that maximize the matching rate by comparing all combinations of clinical practices shown in the log. Fig 3 shows the result that helps us to find the optimal clinical order set. A clinical order with the highest application rate of 99.64% (i.e., 35605373 (remifentanil 1 MG)) is included in the temporary CP set. As a result of this step, the matching rate slightly increases to 50.92%. By iteratively performing this process, the CP is finally configured with 39 orders, and its application rate, matched ratio, and the matching rate reach 44.17%, 38.73%, and 82.90%, respectively.

Table 6 provides the details of the TLH CP consisting of 39 orders. Through the CDM, we obtained clinical orders with standardized concept IDs defined numerically. The detailed description of each order from the CDM vocabulary was added in parentheses.

**Table 6:** Details of the derived CP by order day.

Day	Table	Concept_ID (Concept_Description)
**1 day before**	Drug	925043(bisacodyl 5 MG), 939871(sodium phosphate), 35605994(cefotetan 1000 MG), 42918203(1000 ML Calcium Chloride 0.2 MG/ML / Lactate 3.1 MG/ML / Potassium Chloride 0.3 MG/ML / Sodium Chloride 6 MG/ML Injectable Solution)
**OP day**	Drug	717165(neostigmine methylsulfate 0.5 MG/ML Injectable Solution), 963353(glycopyrrolate), 19077241(famotidine 20 MG Oral Tablet), 35603429(fentanyl 0.05 MG/ML Injection), 35605373(remifentanil 1 MG), 35605994(cefotetan 1000 MG), 36249738(1000 ML glucose 50 MG/ML Injection), 40221385(100 ML sodium chloride 9 MG/ML Injection), 40221390(20 ML sodium chloride 9 MG/ML Injection), 42918203(1000 ML Calcium Chloride 0.2 MG/ML / Lactate 3.1 MG/ML / Potassium Chloride 0.3 MG/ML / Sodium Chloride 6 MG/ML Injectable Solution), 42921670(3 ML Midazolam 1 MG/ML Injectable Solution), 42922137(2 ML Acetylcysteine 150 MG/ML Injectable Solution), 42960507(ramosetron hydrochloride Injectable Solution), 43291560(5 ML Rocuronium 10 MG/ML Injection), 46287423(ketorolac tromethamine 30 MG/ML Injection)
**1 day after**	Drug	19077241(famotidine 20 MG Oral Tablet), 35605994(cefotetan 1000 MG), 36249738(1000 ML glucose 50 MG/ML Injection), 42922137(2 ML Acetylcysteine 150 MG/ML Injectable Solution)
	Measurement	3000666(Metamyelocytes/100 leukocytes in Blood by Manual count), 3007591(Band form neutrophils/100 leukocytes in Blood by Manual count), 3009261(Glucose [Presence] in Urine by Test strip), 3011587(Promyelocytes/100 leukocytes in Blood by Manual count), 3018229(Myelocytes/100 leukocytes in Blood by Manual count), 3021589(Normoblasts [#/volume] in Blood), 3023643(Blasts/100 leukocytes in Blood by Manual count), 3028893(Ketones [Presence] in Urine), 3029937(Albumin [Presence] in Urine by Test strip), 3035124(Erythrocytes [#/area] in Urine sediment by Microscopy high power field), 3035583(Leukocytes [#/area] in Urine sediment by Microscopy high power field), 3037234(Variant lymphocytes/100 leukocytes in Blood by Manual count), 3041543(Other cells/100 leukocytes in Blood by Manual count), 40758562(Immature cells/100 leukocytes in Blood), 40761899(Leukocytes [#/volume] in Urine by Automated test strip)
**2 days after**	Drug	19086516(aceclofenac 100 MG)

To examine the difference of CDM-derived CP, we compare the models from CDM with those from typical EHR data. The existing EHR-based clinical log was composed of multiple different clinical orders that can be grouped into a single target concept in the CDM. For example, 19077241 (i.e., famotidine 20 MG Oral Tablet), was utilized in three different clinical orders: FMTD, FMTDD, and FMTDI. It is found that in the CDM-derived CP we can take advantage of the coarser-grained level of orders when performing process analysis. The standardized vocabulary in CDM also enables analysis using higher-level data such as ingredient or Anatomical Therapeutic Chemical (ATC) classification, according to medical professionals.

Applying the proposed method, we extract the order log for additional surgical procedures: THR, CB, TAVI, and PD. Overall, we follow the query suggested in S1 Table, except we use 1) five different procedure_concept_ids for CB and 2) ancestor_concept_id instead of procedure_concept_id for PD due to the limited number of cases extracted when procedure_concept_id is applied. Table 7 shows the summary of the extracted datasets.

**Table 7:** Summary of the datasets.

	THR	CB	TAVI	PD
**Number of cases**	1,022	458	31	235
**Average number of measurements**	191	985	487.8	543.1
**Average number of medications**	68.3	271	127.9	215.7
**Average LOS**	5.8	13.3	9.2	13.8
**Source of admission**	Outpatient: 988	Out: 294	Out: 26	Out: 212
	ER: 13	ER: 161	ER: 5	ER: 23
	Other: 21	Other: 3		Other: 1
**Discharge destination**	Home: 868	Home: 444	Home: 29	Home: 232
	Transfer: 63	Transfer: 5	Transfer: 1	Transfer: 3
	Nursing: 7	Death: 6	Death: 1	Nursing: 1
	Other: 84	Nursing: 2		
		Other: 1		

**Fig 4:**
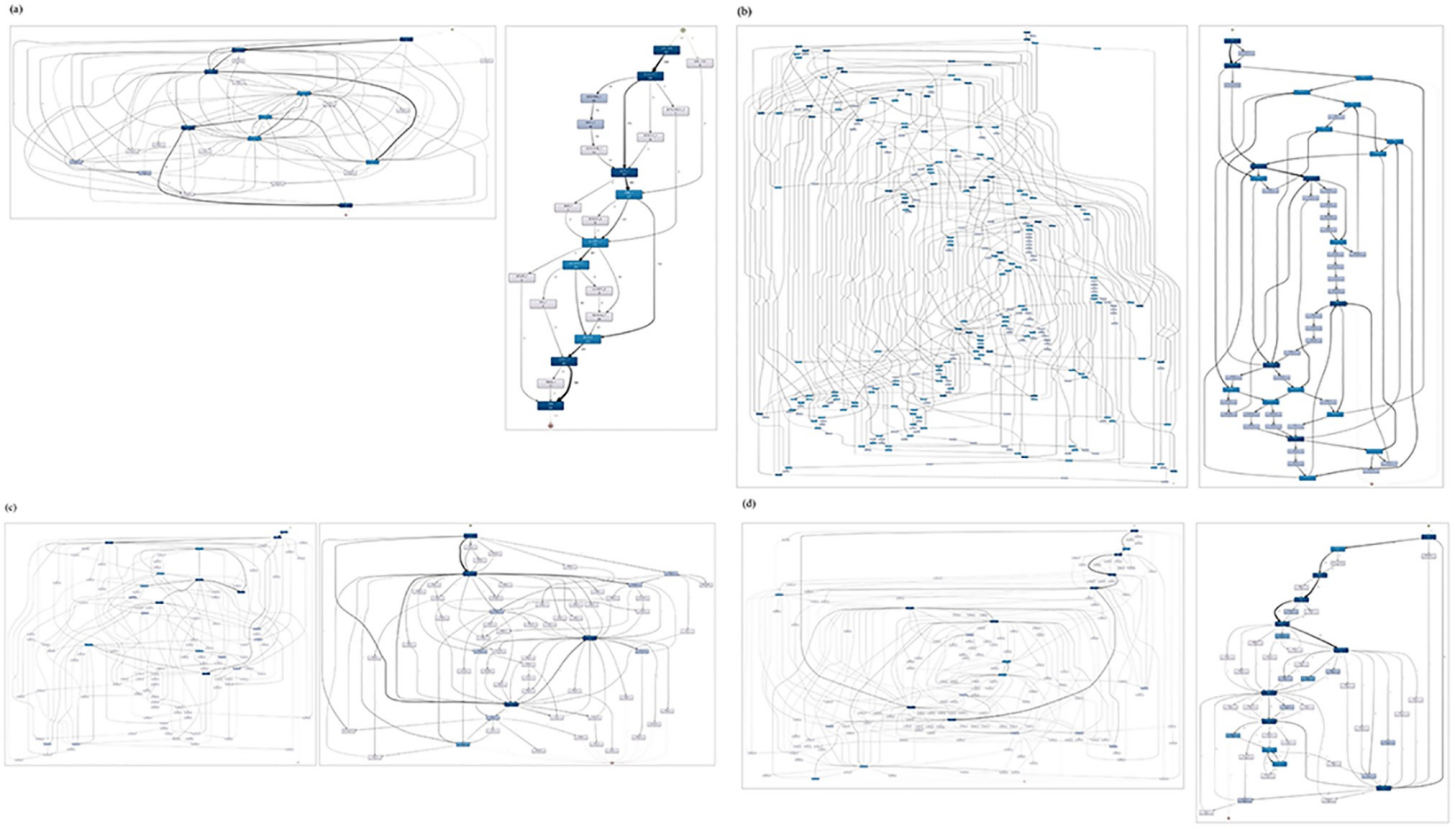
CP discovery for selected surgeries. (From left to right) (a): CP before THR for mainstream behaviors only with all paths, CP before THR with mainstream behaviors and major paths only; (b): CP after CB for death cases, CP for 24 hours after CB for death cases; (c) CP before TAVI, CP for 24 hours after TAVI; (d): CP for 24 hours after PD with all activities, CP for 24 hours after PD with 30% activities.

Given the event log formulated, we discover and demonstrate CPs in Fig 4, which can serve as a baseline for further analysis, such as CP variance management, temporal pattern mining, and personalized CP generation. In addition, we apply different time windows as suggested in [16, 17] and filter events with higher frequencies only due to the variety of clinical orders and their repetitive occurrences. Overall, the processes are highly fragmented and customized to each patient case. An enlarged view of each process model is shown in S1 Fig.

### Outpatient process

**Fig 5:**
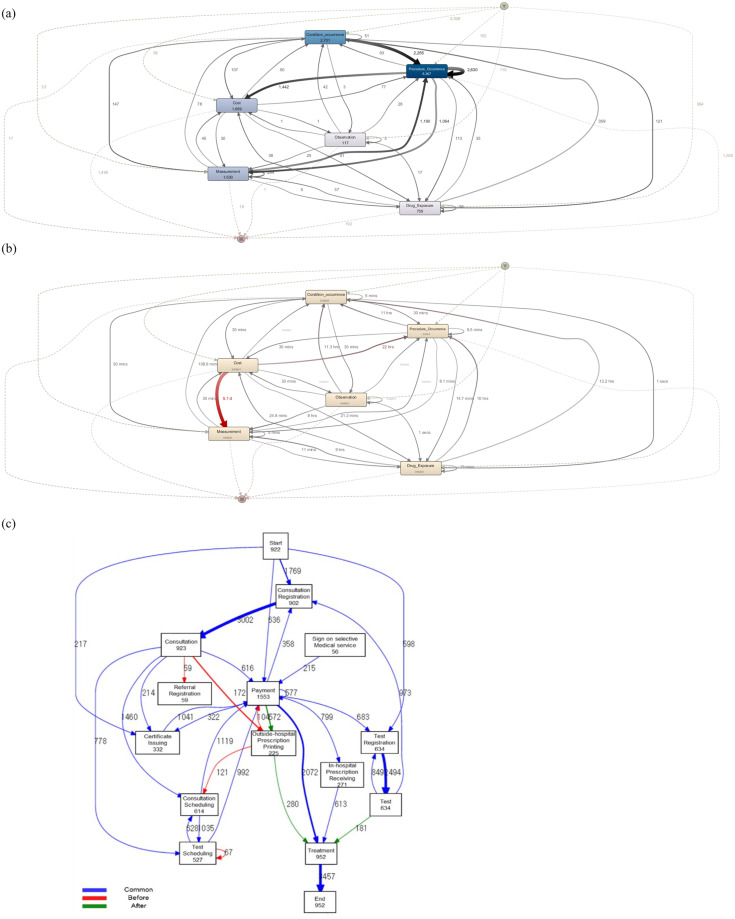
Outpatient process models. (a) CDM-based model in frequency view; (b) CDM-based model in performance view; (c) EHR-based model in performance view [42].

In this section, we focus on the comparison between the CDM-based process model and the existing EHR-based process model using our outpatient event log. We found that CDM-based outpatient process models can exhibit a different pattern from EHR-based models. Fig 5(a) and 5(b) show process models discovered from our CDM dataset. The most frequent event is *procedure*, followed by *condition*, *cost*, *measurement*, *drug*, and *observation*, and the path with the highest frequency begins with *condition* and proceeds to two subsequent *procedures*. *Measurement* and *observation* are rarely observed. On the contrary, Fig 5(c), which is an EHR-based process model excerpted from [42], shows a more complicated pattern due to the more exhaustive representation of the included activities. The total number of activities is 12, including registration for consultation, referral, test, sign-on selective medical service, certificate issuing, scheduling for consultation, and test. These activities are rarely observed in our CDM dataset, resulting in a simplified outpatient process model.

**Fig 6:**
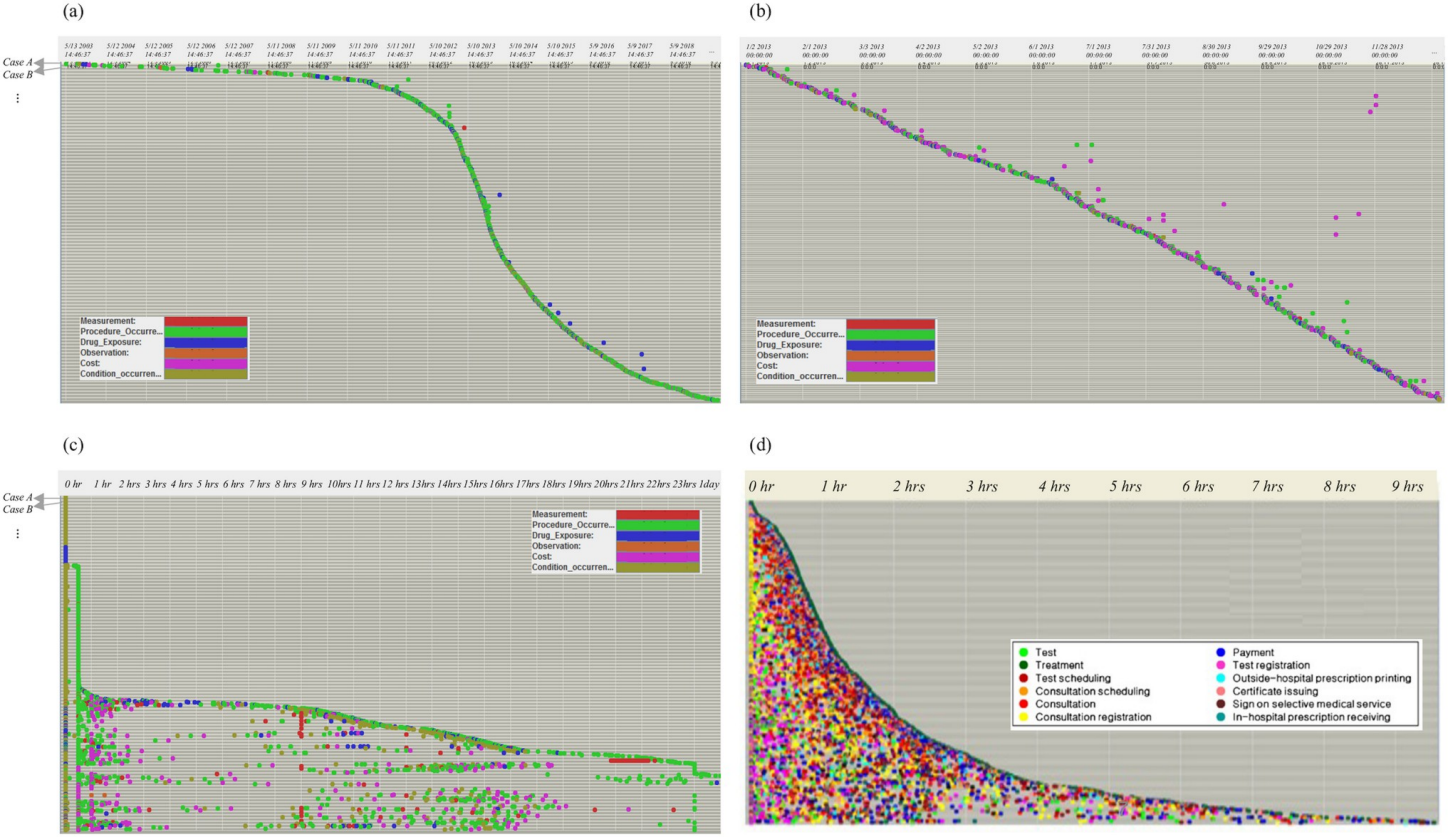
Dotted chart analysis. (a) dotted chart for all activities by case, occurred between 2003 and 2019; (b) dotted chart for activities by case, occurred in 2013; (c) dotted chart for all activities occurred by case, sorted by case duration; (d) dotted chart for all activities occurred by case, sorted by case duration, excerpted from [42].

This pattern is also observed in dotted chart analysis, one of the popular process mining tools that provides a holistic view of the entire process and allows us to identify activity patterns at different levels of temporal granularity. Fig 6(a) shows the occurrence of six activities of each case (i.e., Y-axis) over time (i.e., X-axis), sorted by case start time. Since our dataset contains the cases of TLH patients, all instances include procedure (i.e., inclusion event), the activity indicating either an operation, treatment, or a radiology test. We can also observe that a procedure is frequently followed by a drug, indicating a prescription of medication after an operation. Fig 6(b) shows events that occurred in 2013 only, where we can observe relatively lengthy cases with delayed payments. In Fig 6(c), the cases (i.e., X-axis) are sorted by case duration (i.e., Y-axis). It is found that almost half of the cases contain one or two activities that occur in a short period, whereas the other half exhibit a relatively widespread pattern with the six activities. Several procedures are observed around the center of the graph, the description of which cannot be identified; that is, whether they indicate an operation, a treatment, or a radiology test is unknown due to the model’s coarse-grained activity representation, in contrast to the dense data points in Fig 6(d). The result of an outpatient process analysis heavily relies upon the abstraction level of administrative data, often characterized by low accuracy and high level as specified in [52].

### Emergency room process

**Fig 7:**
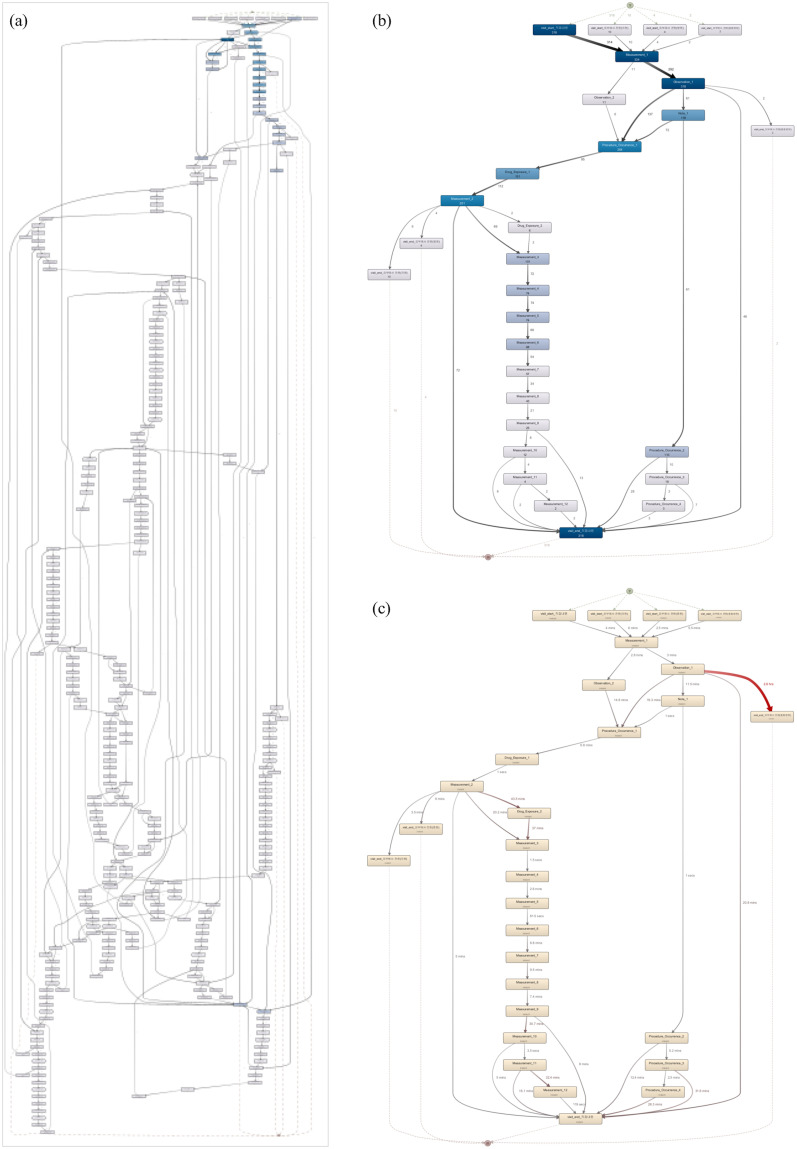
Emergency room process. (a) Process model with all activities included; (b) Process model with mainstream behaviors only; (c) Process model with mainstream behaviors only, based on median time duration between events.

Fig 7 shows the discovered process models from the ER event log. Although we use the selected CDM table names as activities, we can still delve into measurements, drug_exposure, and notes for more detailed information (e.g., Note_chest AP). The sources of admission and discharge are also annotated in the event names. Although the models do not provide administrative details such as registration and payment, the models help get a grasp of specific clinical interventions during ER visits and the average time taken between activities. The most frequent trace, shared by 33 cases, begins with an admission from home, followed by measurement, observation, and discharge to home.

### Patient journey

**Fig 8:**
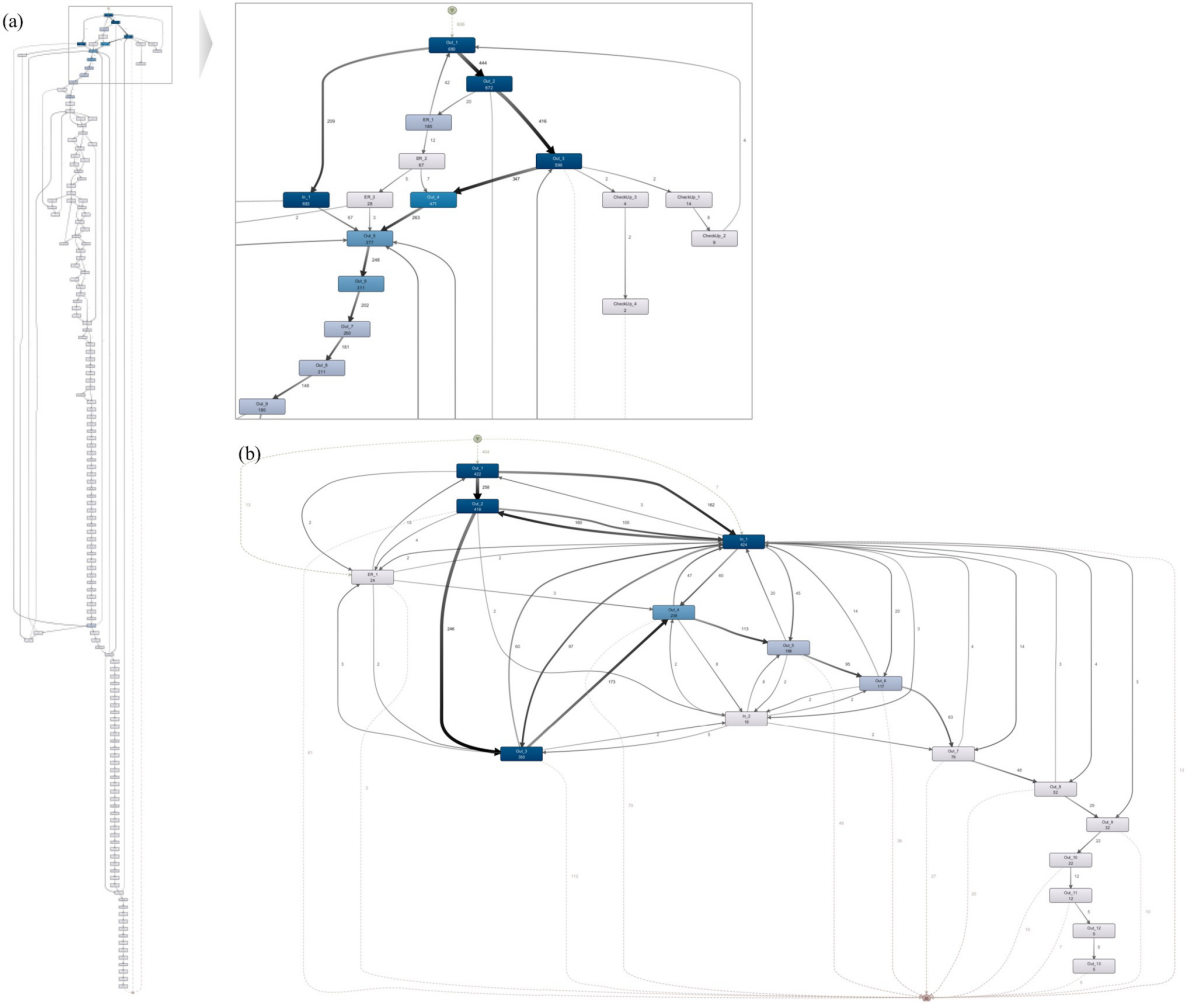
Patient journey map using visit-level events. (a) entire patient journey with all activities included (left) and the enlarged screenshot of the early encounters only (right); (b) patient journey with mainstream behaviors only.

We also discover patient journey maps on two levels based on the CDM-derived event logs. Fig 8 shows process maps based on the ordered patient encounters for the selected time window, i.e., January 2017 to December 2020. The maximum number of visits is 117, 23, 9, and 4 for outpatient, inpatient, ER, and checkup visits, respectively. The most frequent path is Out_1 > In_1 > Out_2 > Out_3 shared by approximately nine percent of all patients (i.e., 62 cases), indicating an outpatient visit before surgery, followed by a hospitalization for the surgery and two subsequent outpatient visits to monitor the patient’s condition. The outpatient visits after the surgery occur one or two weeks after the admission date and a month after the preceding visit.

The second most frequent path is Out_1 > In_1 > Out_2, shared by eight percent of all cases, indicating that some patients visit the hospital after the operation only once. For these two variants, the elapsed time between the very first outpatient visit and the subsequent inpatient visit is 42.4 days on average. After that, it takes approximately 11 days until the next outpatient visit occurs (median duration). The checkup visits occur for medical examination only and are not frequently observed in our dataset. However, detailed information on each activity type is available (e.g., Measurement_ABDO, Procedure_Occurrence_H0651). The most frequent activity during the checkup visits is ‘Measurement’, followed by ‘Procedure_Occurrence’ and ‘Drug_Exposure.’

**Fig 9:**
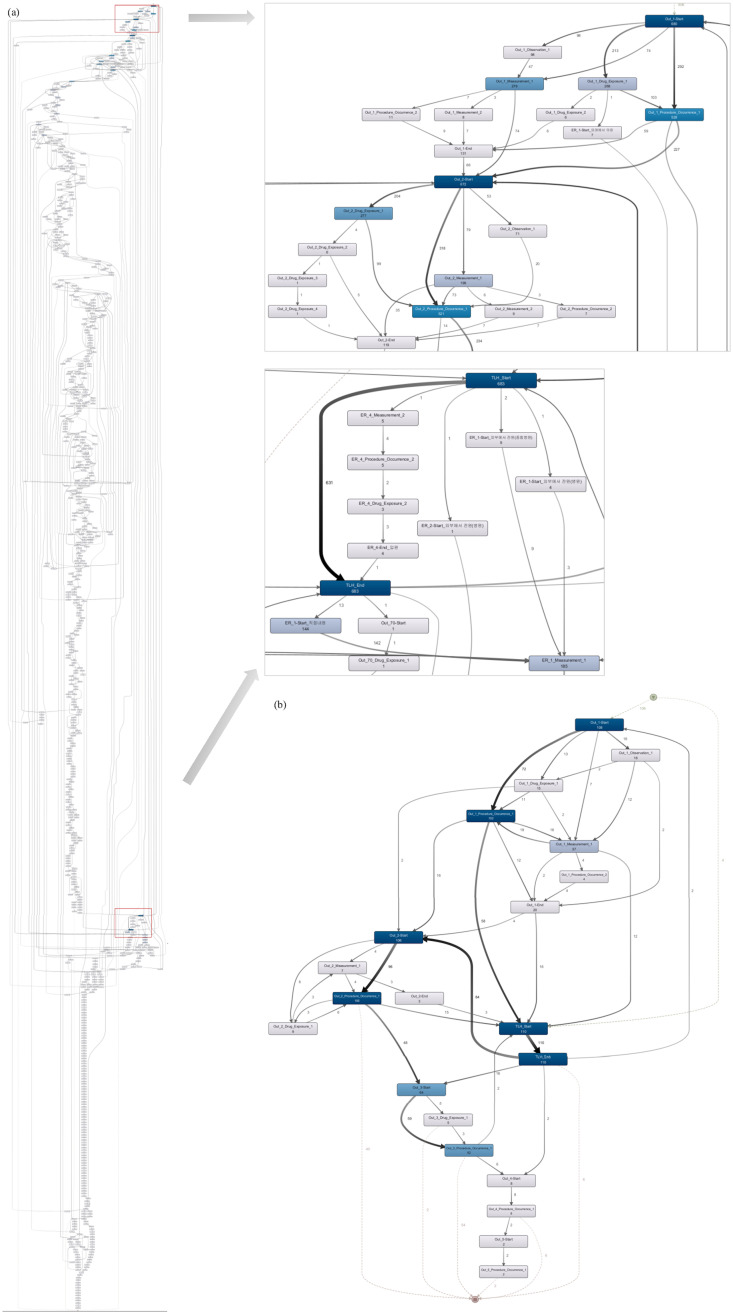
Patient journey map using descendent-level events. (a) process model for the entire visits (left); enlarged view of the first two outpatient visits (top right); enlarged view of the TLH inpatient visit (middle right) (b) process model with mainstream behaviors only.

Now, we examine descendent-level activities during each encounter, as shown in Fig 9. Although individual clinical orders are available for each hospitalization day, we save this information for the inpatient journey for a recognizable patient journey. For individual orders and detailed medication codes, a more detailed version of the model can be obtained. To observe the visit pattern clearly, we append synthetic ‘start’ and ‘end’ for each process type (e.g., Out_1_Start).

Fig 9 shows the descendent-level map, a fine-grained version of the models in Fig 8. For simplification, the same activity types that occurred on the same date have been removed, and the remaining recurring activities for outpatient, ER, and checkup visits have been given indices by order of occurrence. The two process models on the right in Fig 9(a) show 1) the flow in the first two outpatient encounters (i.e., Out_1, Out_2) and 2) events intertwined with the first inpatient visit. Given the descendent-level map, we can observe intervention patterns during each visit in addition to the visit pattern identified in the visit-level map.

## Discussion and future work

Overall, CDM-based event logs are as competent as EHR-based logs in identifying the most followed and exceptional paths (RQ 1). In addition, as shown in the inpatient analysis for the selected surgical procedures, event log for different patient groups can be easily extracted from CDM and compared using the common queries even in different sites (RQ 2). Here, the patient groups can also be defined based on patient outcomes logged in CDM, e.g., discharge to home, transfer, or death, in place of particular operations or diagnoses. However, as in EHR-based process mining, to check whether the model complies with internal or external guidelines (RQ 3), we would still need additional resources such as reference CP for each diagnosis or COVID-19 treatment guidelines. Bottlenecks can also be easily identified (RQ 4) by applying the performance perspective to the discovered models. However, it should be noted that the lack of representation of operational activities in CDM may limit the model’s ability to answer RQ 1 and RQ 4 with the best accuracy for outpatient processes. For example, if the model shows that it takes a long between procedure and drug, that would not provide much insight into enhancing the process due to the activities’ high abstraction levels.

**Fig 10:**
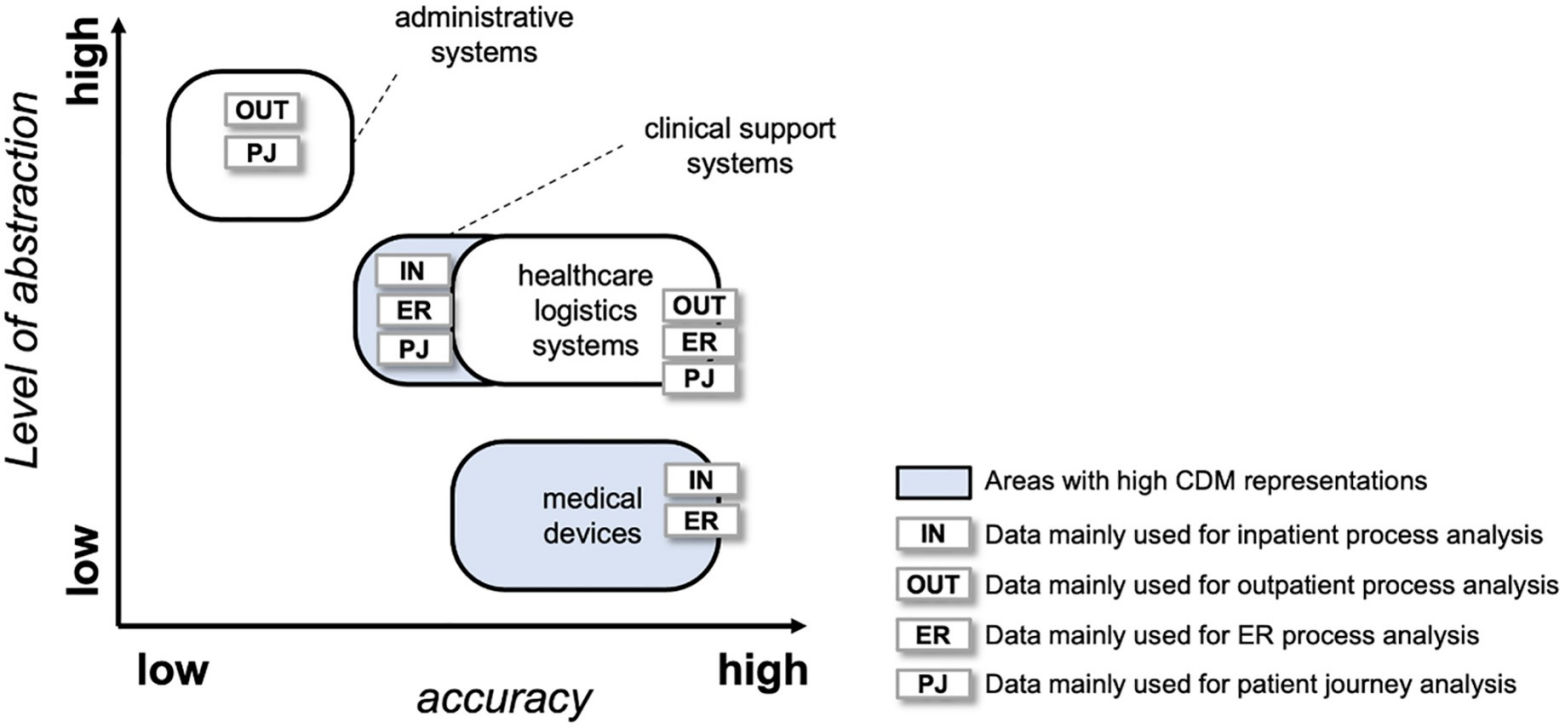
Process mining data spectrum and representation ability of CDM, based on [52].

As of now, CDM does not fully cover the entire process mining data spectrum proposed by [52]. As depicted in Fig 10, CDM has high representations of data from clinical support systems and from medical devices, but low representations of data from administrative systems and healthcare logistics systems. Until the transformation of such data become supported by and available in CDM, CDM-based event logs should be complemented by data from different internal and external sources.

Regarding CP applications in inpatient process analysis, we observe that clinical orders can be more efficiently managed by adopting CDM. As explained earlier, CDM provides clinical orders at multiple levels of granularity, which enables the discovery of both rough and comprehensive CP models for additional insights from the models. Overall, discovering and analyzing an inpatient process within a CDM environment is relatively straightforward compared to the other processes, due to the CDM’s high representation of clinical observational data. As of now, however, current CDMs do not incorporate nursing interventions and diet information, which can be appended to CP. This limitation would be overcome in the future as the conversion process evolves. In addition, even within a CDM environment, knowledge-based CPs by domain experts are still required for conformance checking and for the evaluation of the derived model.

Given the additional CPs discovered for selected surgeries, we identify the differences between our case studies and the existing studies, as shown in Table 8. We included all four surgeries discussed in both studies, adjusted the date range to approximate the total number of patients selected in the studies and aligned the time windows. Although we could use patient location as activities for our case study, we use clinical orders instead to show more detailed CP models due to the sparsity of location data.

**Table 8:** Comparison with other studies.

	This study	[16]	[17]
**Target surgeries**	THR, CB, TAVI, PD	THR, CB, TAVI	PD
**Target patients**	1,022; 458; 31; 235	1,013; 566; 357	551
**Date range**	4 years (2018 ~ 2021)	1 year (2019)	9 years (2010 ~2018)
**Time window**	THR: from admission to operation	THR: from admission to operation	PD: from operation to discharge
	CB: from operation to death	CB: from operation to death	
	TAVI: from admission to discharge	TAVI: from admission to discharge	
	PD: from operation to discharge		
**Data source**	CDM event log	CDM event log	EHR event log
**Activities**	Clinical orders	Patient location	Patient location
**Visualization**	Process models	Sankey diagrams	Sankey diagram

From our CDM-based process analysis, we have discovered a few findings. First, our process models can present more process-related insights, e.g., the absolute frequency of each event and descriptive statistics of times taken between events, compared to the Sankey diagrams shown in [16, 17]. Second, we can adjust the granularity of the activity values with the advanced degree of freedom, which enables us to conduct multi-level CP discovery. CDM provides source values for a wide range of events that occur at hospitals, e.g., visits, observations, measurements, and procedures. With CDM, such events can be delved into without requesting additional data. Lastly, our results open the door for further process analysis beyond visualization since model discovery is considered the starting point of process mining. Without process mining perspectives, treatment pathways could merely be one of the visualization tools.

We have also received positive feedback from medical professionals on the results, in that the process mining enables the researchers to easily adjust abstraction levels for more detailed process-related information while providing a holistic view of the entire process. We have also been told that the process mining-based visualization is more effective in obtaining process-related knowledge than visualization tools provided by OHDSI (e.g., Sankey diagrams, sunburst plots).

Outpatient and ER processes should be able to provide operational perspectives using administrative events ranging from registration to payments. However, the CDM dataset used in this paper does not provide such information. For this reason, there can be limitations in conducting performance analysis related to patient satisfaction and hospital operational efficiency (e.g., waiting time from registration to consultation for outpatients). Thus, a future research agenda remains to include operational events sufficiently detailed in CDM. Although there is a concept for ‘patient registration’ (Concept ID 40318361), CDM still needs vocabulary to map operational activities such as test registration, consultation start and end, referral registration, consultation scheduling, prescription printing, and certificate issuance to be complemented with process mining perspectives.

To this end, according to medical professionals, the CDM structure should be extended to cover more administrative data mapped from the EHR. One of the considerations prior to extending the CDM structure is the information on each site’s CDM mapping. The expressive power of CDM, however, can be different across organizations depending upon the vocabulary mapping level and the resultant data quality. To address this issue, OHDSI provides ETL conversion rules and is continuously updating the rules. In addition, CDM users should deliberately review information on the details of data mapping, which should be governed by domain-specific professional guidelines, before the CDM content is used for comparison or integration with data from other sources. This would decrease the possibility that the value creation of analysis is hindered by CDM’s data quality.

Through patient journey analysis, we can obtain a holistic view of what has occurred to a particular patient or patient cohort. The multiple levels of granularity, volume, and precision of clinical data offered by the CDM can help expedite the undertakings of such analysis. The medical professionals also noted that, in the future, it would be worthwhile for researchers to compare patients’ journeys with the same diagnosis from different hospitals.

The CDM allows for an environment in which clinical logs from multiple care sites are integrated by applying similar algorithms in a distributed setting. To this end, we propose a few future directions for CDM-based process mining in healthcare. First, the CDM would allow a direct process model comparison between hospitals. This comparison would in turn enable the evaluation of healthcare workflows and process improvement in each site. Moreover, data obtained from multiple sites can serve as a baseline in constructing a global reference process model. It would no longer be challenging to build such a model after the data structure of each care site were aligned. In addition, we would also be able to collect relevant parameters from each site to update the global model in the manner of federated learning. Further, the CDM would facilitate research on personalized healthcare by integrating care delivery records (i.e., patient journeys) of the same patient from different sites.

## Conclusion

This paper proposes a method to locate, define, and extract event logs within a CDM environment for healthcare process mining and validates its usability with real-life cases by demonstrating process analysis for different process types. Most of the existing studies on the CDM have not paid much attention to process mining, concentrating either on the transformation of the EHR data to CDM or on clinical outcome predictions based on machine learning. As a novel attempt to exploit CDM for process mining, we demonstrate event log extraction and perform process analysis using the CDM-extracted event log.

The contributions of this paper are as follows. First, we provide a step-by-step guidance for process mining researchers to extract and build event logs from CDM. This is crucial since this method can be generalized to different hospitals, owing to the standardized nature of the database. Second, we demonstrate different types of process analysis using CDM, the data source that is becoming a new standard in healthcare research. We also compare the results of CDM-based process analysis and EHR-based analysis, to suggest considerations when conducting CDM-based research. In addition, as noted earlier, the conversion to CDM opens an opportunity to meet process mining challenges. This work can serve as a starting point in resolving such challenges. The ability to leverage the CDM, which is still an untapped reservoir of healthcare data, can be a valuable asset in efficiently and effectively deriving process-related knowledge. Lastly, for existing CDM users who engage in CDM-based healthcare research in general, we demonstrate process mining to introduce it as a useful tool in analyzing complex hospital processes including clinical and non-clinical activities. This can contribute to bringing a process mining perspective to the changing HIS environment and to facilitating research on data mapping and conversion for process analysis.

## Supporting information

S1 Table The query for event log formulation. (PDF)

S1 Fig Enlarged view of Fig 4. (PDF)
