# Supplementary material for: Exploring the potential of OMOP common data model for process mining in healthcare
Source: PLoS One. 2023 Jan 3;18(1):e0279641. doi: 10.1371/journal.pone.0279641 (PMC9810199; doi:10.1371/journal.pone.0279641)
Supplement: S1 Table — (PDF) [file pone.0279641.s001.pdf]

S1 Table. The query for event log formulation.

```
/* get required elements of event logs */
SELECT    Visit.Visit_occurrence_id as CaseID,
              ET.[Table included in Scope]_occurrence_id as EventID,
              ET.[Table included in Scope]_concept_id as Activity,
              ET.[Table included in Scope]_datetime as Timestamp,
              ET.Provider_id as Originator,
              /* specify case attributes */
              [Visit.column_name],
              /* specify event attributes */
              [ET.column_name]
  FROM      ( /* combine Visit_occurrence and Provider tables */
                 SELECT   *
                 FROM      ( /* combine Visit_occurrence and Person tables */
                                SELECT   *
                                FROM Visit_occurrence LEFT JOIN Person
                                ON Visit_occurrence.Person_id = Person.Person_id
                             ) AS Visit_temp LEFT JOIN Provider
                 ON Visit_temp.Provider_id = Provider.Provider_id
              ) AS Visit,
              ( /* combine [Table included in Scope] and Provider tables */
                 SELECT *
                 FROM [Table included in Scope] LEFT JOIN Provider
                 ON [Table included in Scope].Provider_id = Provider.Provider_id
              ) AS ET
  WHERE   /* connect Visit table and ET table */
              Visit.Visit_occurrence_id = ET.Visit_occurrence_id AND
              /* filter with a specific process type */
              Visit.Visit_type_concept_id = [Process type] AND
              /* filter with a specific period */
              Visit.Visit_start_date >= [Period.lowerbound] AND
              Visit.Visit_end_date <= [Period.upperbound]
```

The lines 11 through 24 describe ways in which tables are selected and joined to constitute event logs. In this process, the table that represents case (i.e., *visit*) is constructed by combining *visit_occurrence*, *person*, and *provider* tables as described in lines 11 through 19. For the tables associated with events instead of cases, the *provider* table and the ones mentioned in the scope part should be considered (lines 20 through 24). This process can be performed in a repetitive manner with the UNION ALL statement when multiple tables are defined for scope. Also, the user-defined elements in the process type and period parts are specified in lines 27 through 31. The feature part can be inserted under the WHERE statement after line 31.
