# Supplementary material for: Exploring the potential of OMOP common data model for process mining in healthcare
Source: PLoS One. 2023 Jan 3;18(1):e0279641. doi: 10.1371/journal.pone.0279641 (PMC9810199; doi:10.1371/journal.pone.0279641)

S1 Fig. Enlarged view of Fig 4

(a) (left) CP before THR for mainstream behaviors only with all paths

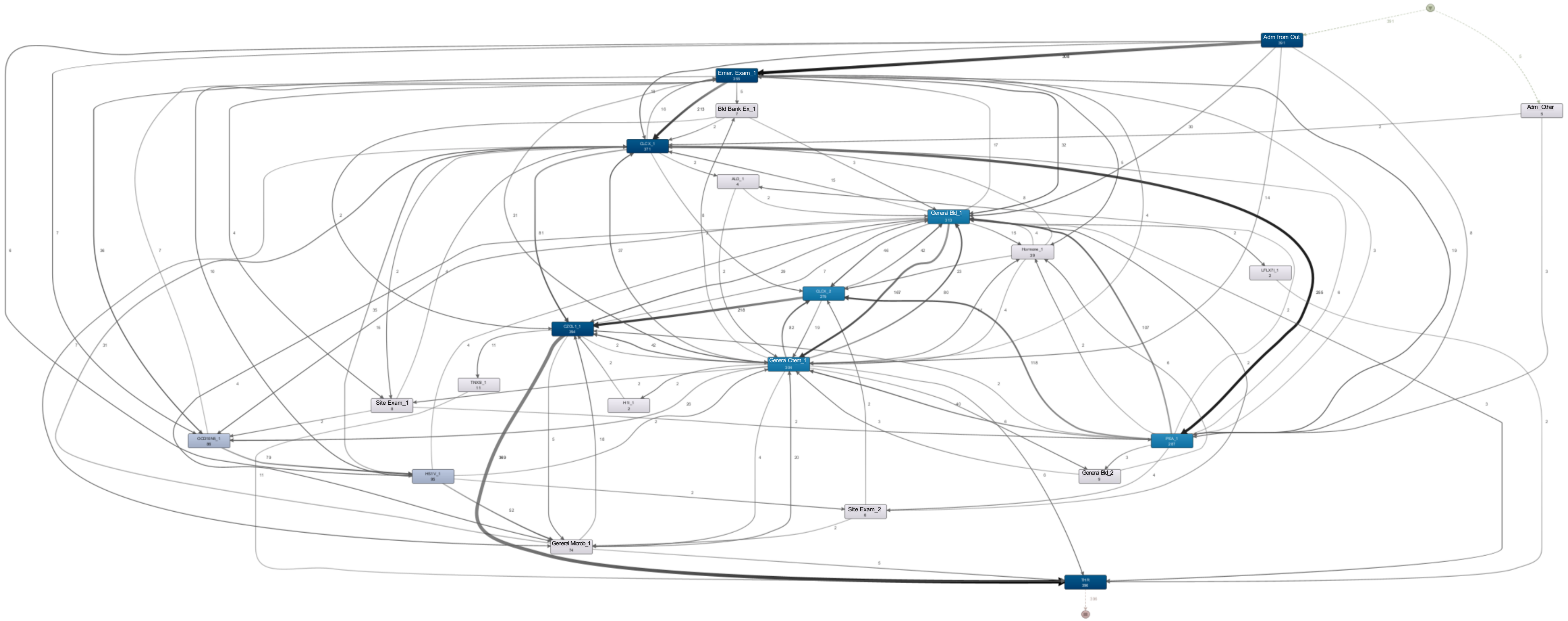

(a) (right) CP before THR with mainstream behaviors and major paths only

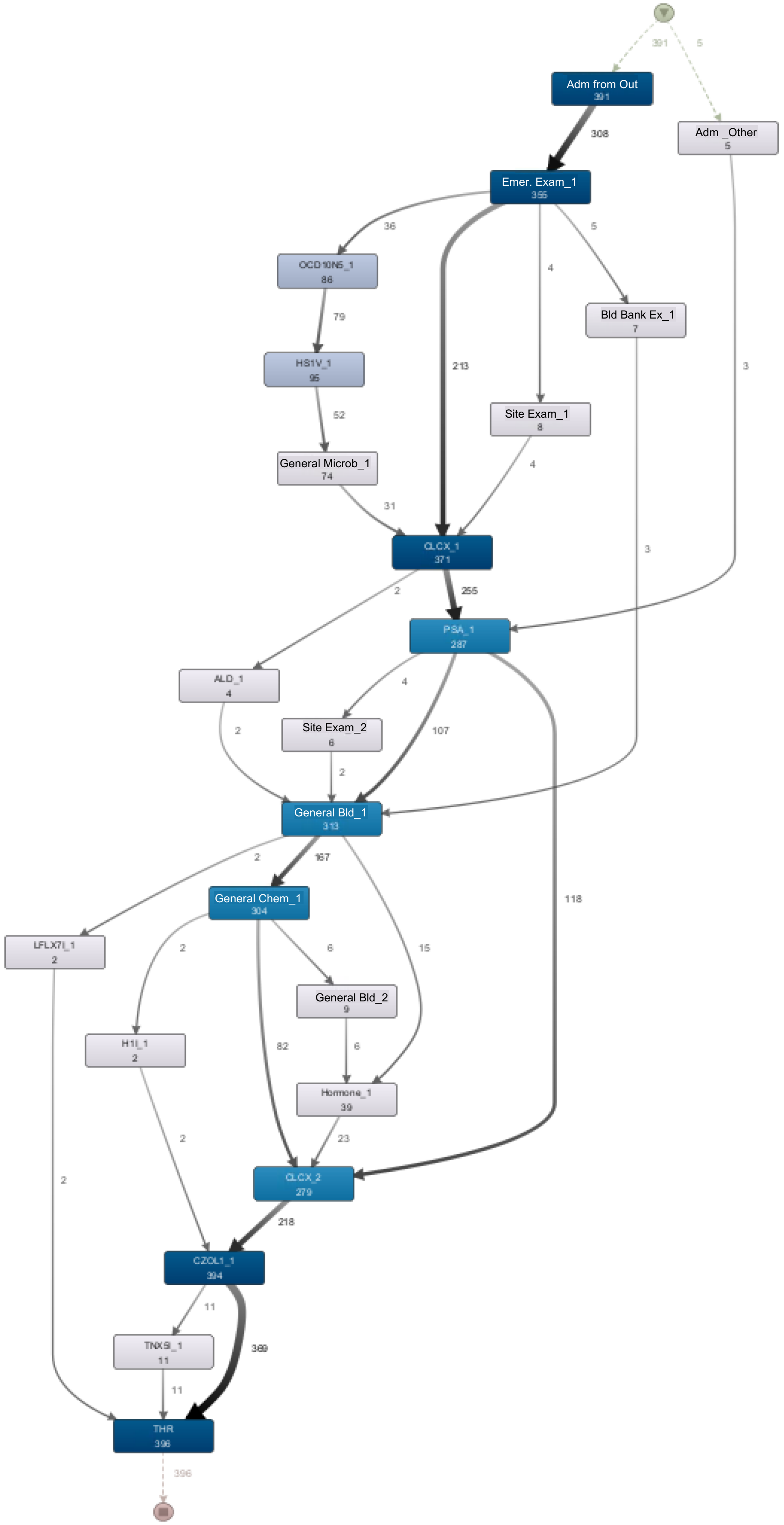

(b) (left) CP after CB for death cases

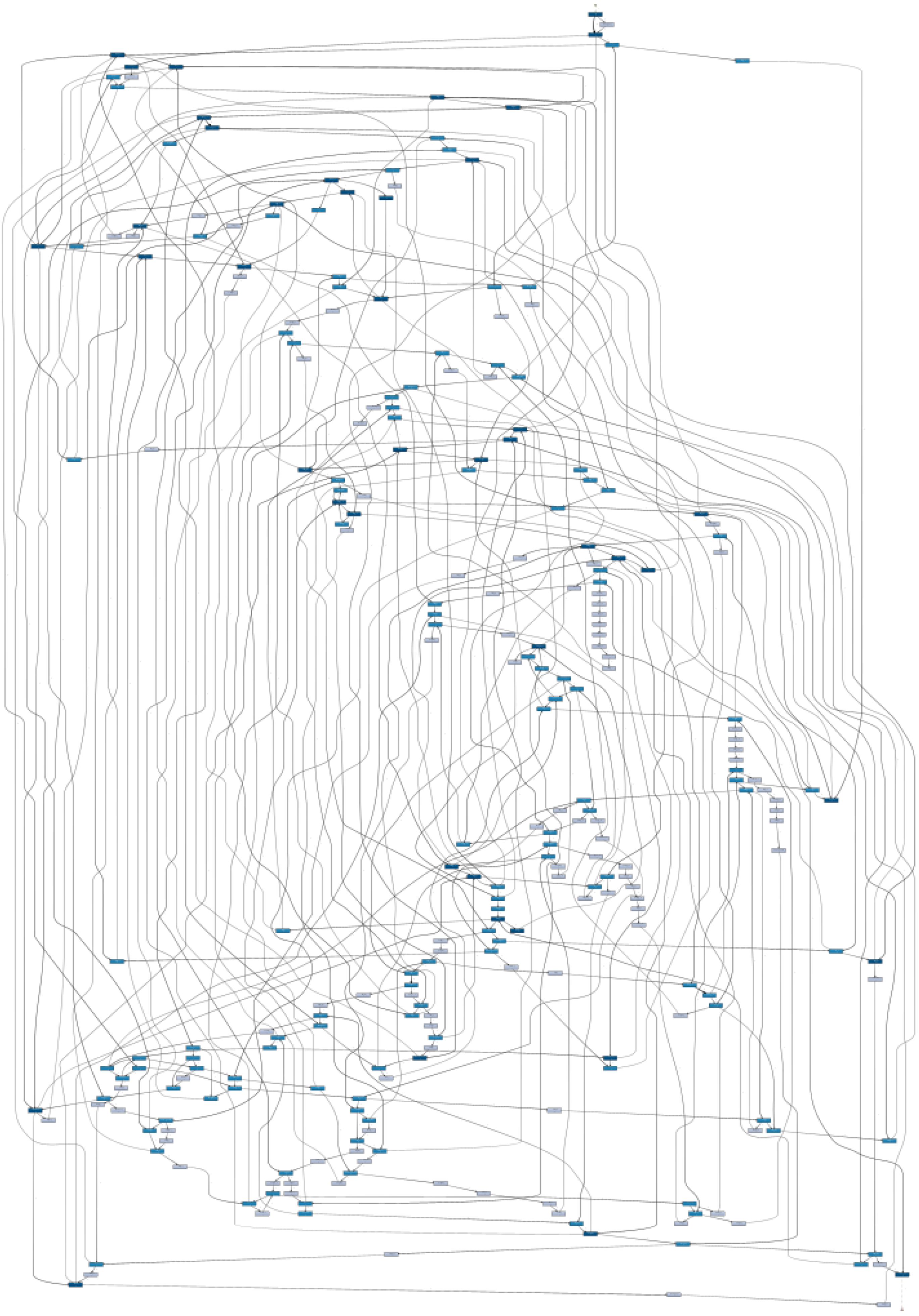

(b) (right) CP for 24 hours after CB for death cases

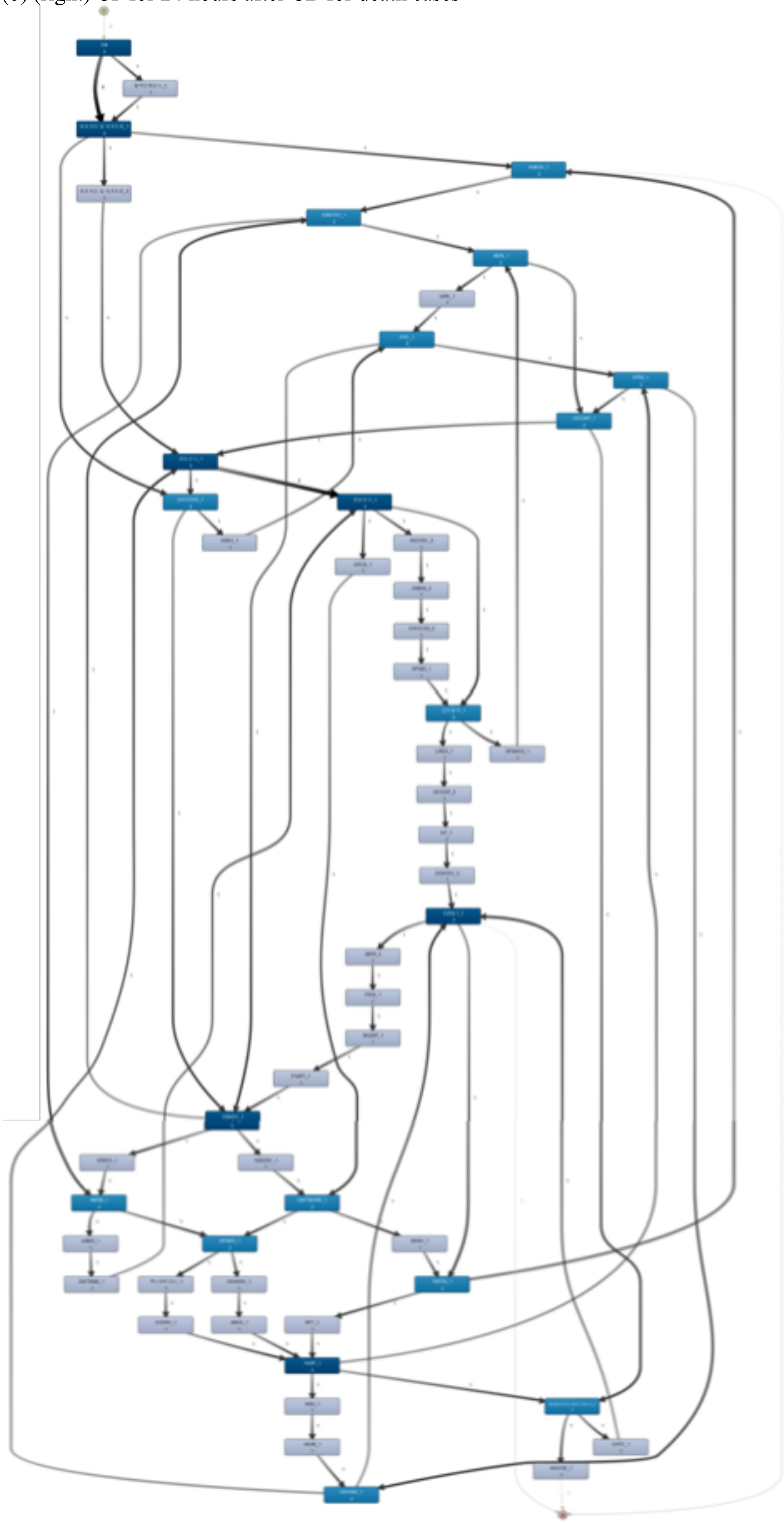

(c) (left) CP before TAVI

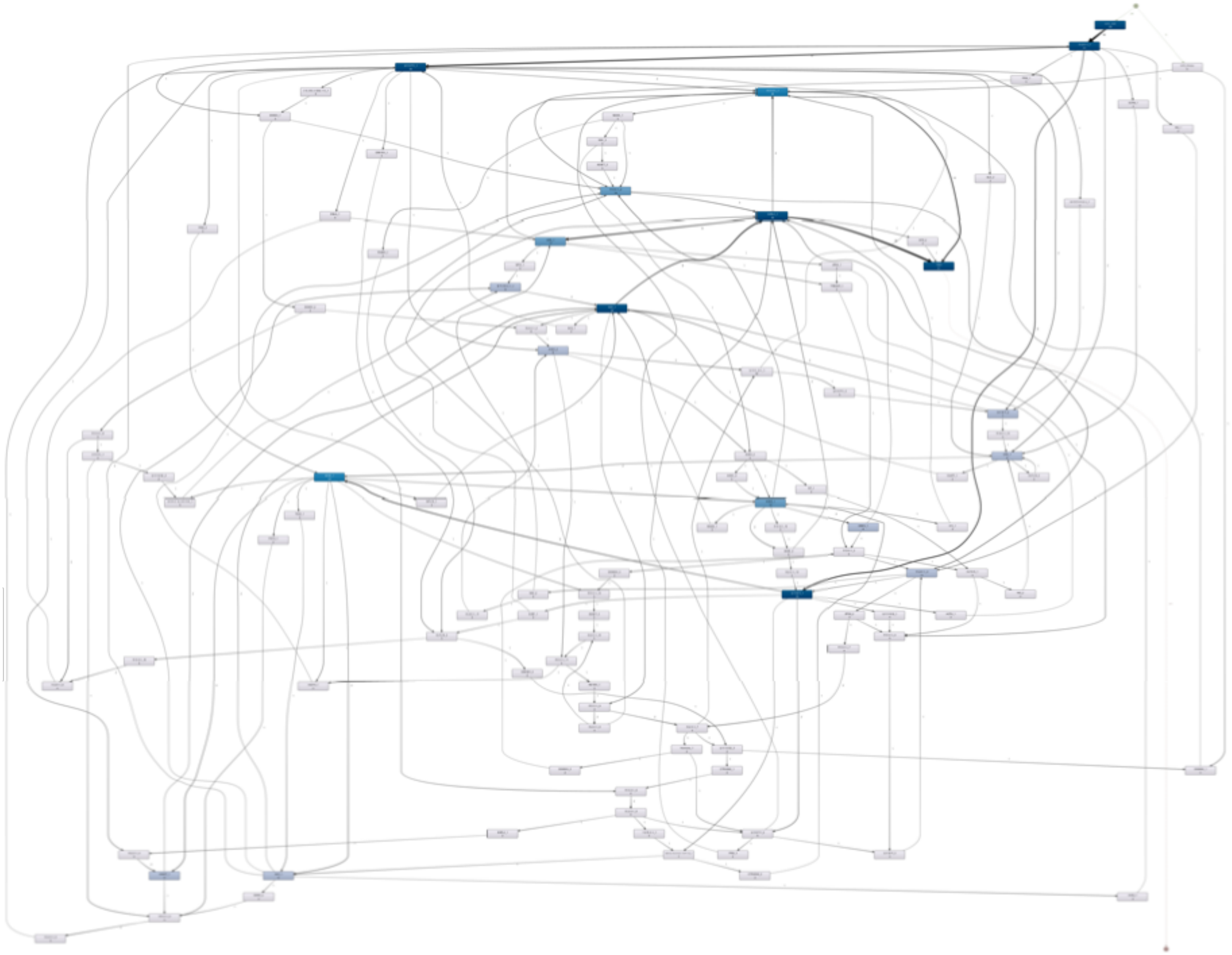

(c) (right) CP for 24 hours after TAVI

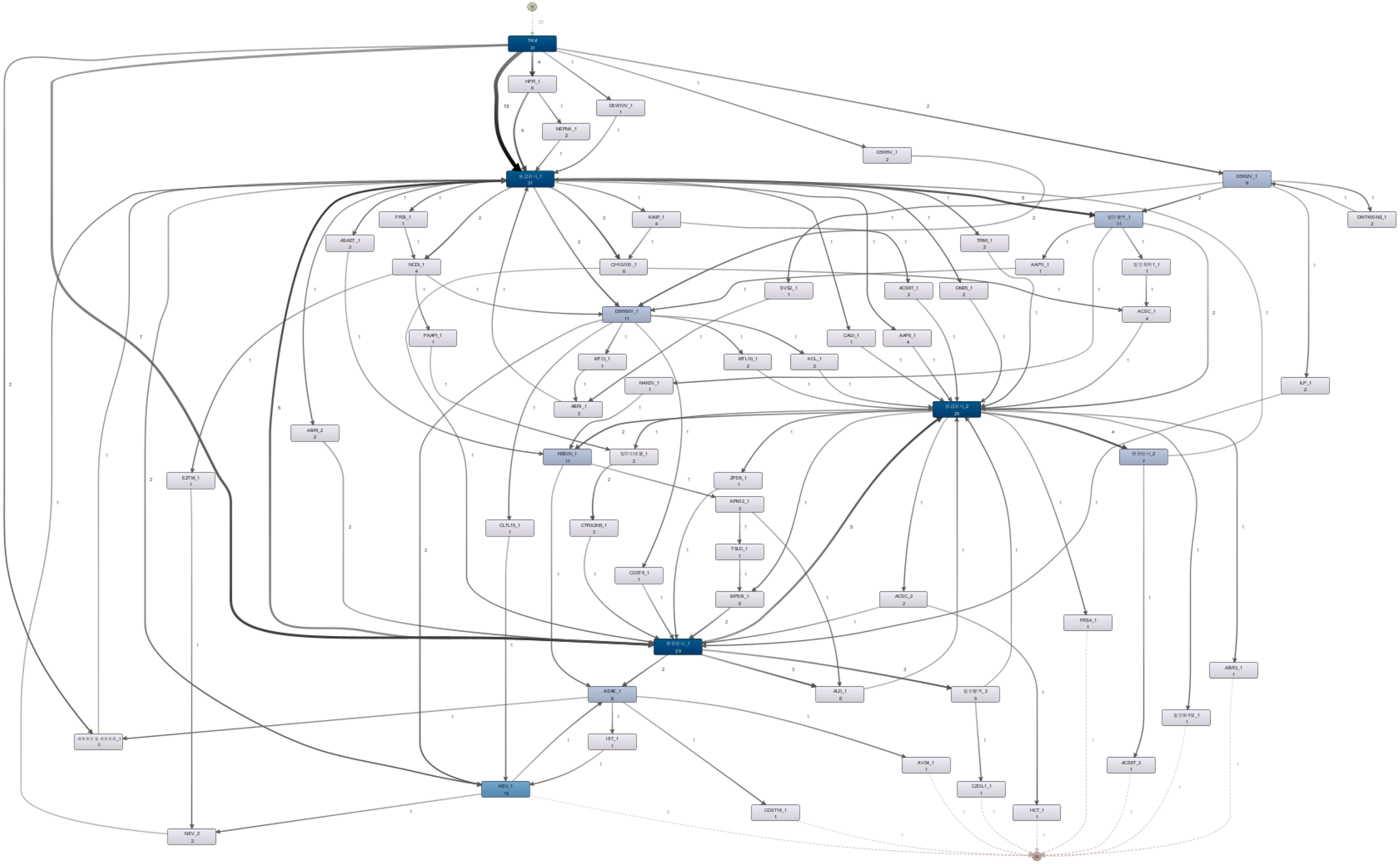

(d) (left) CP for 24 hours after PD with all activities

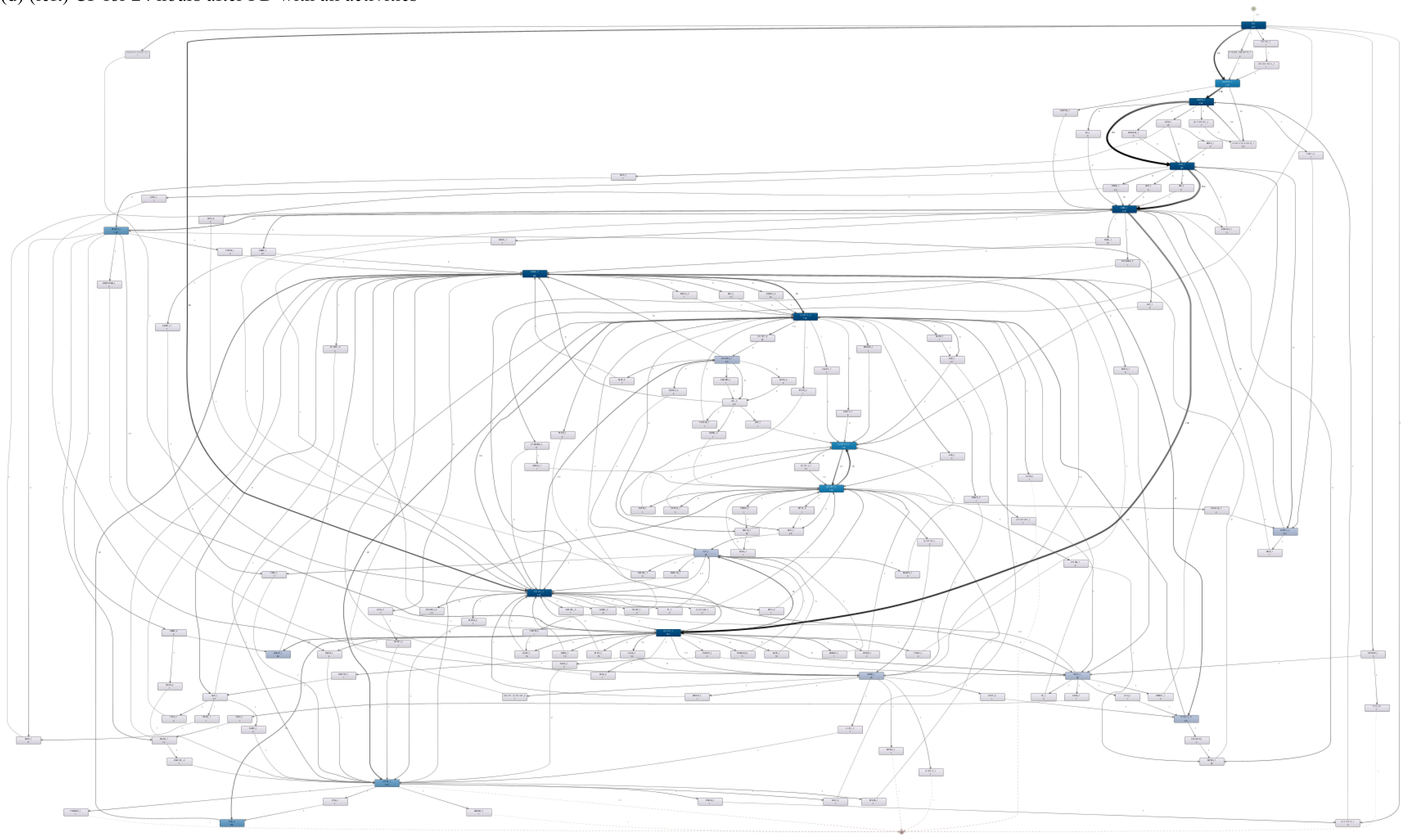

(d) (right) CP for 24 hours after PD with 30% activities

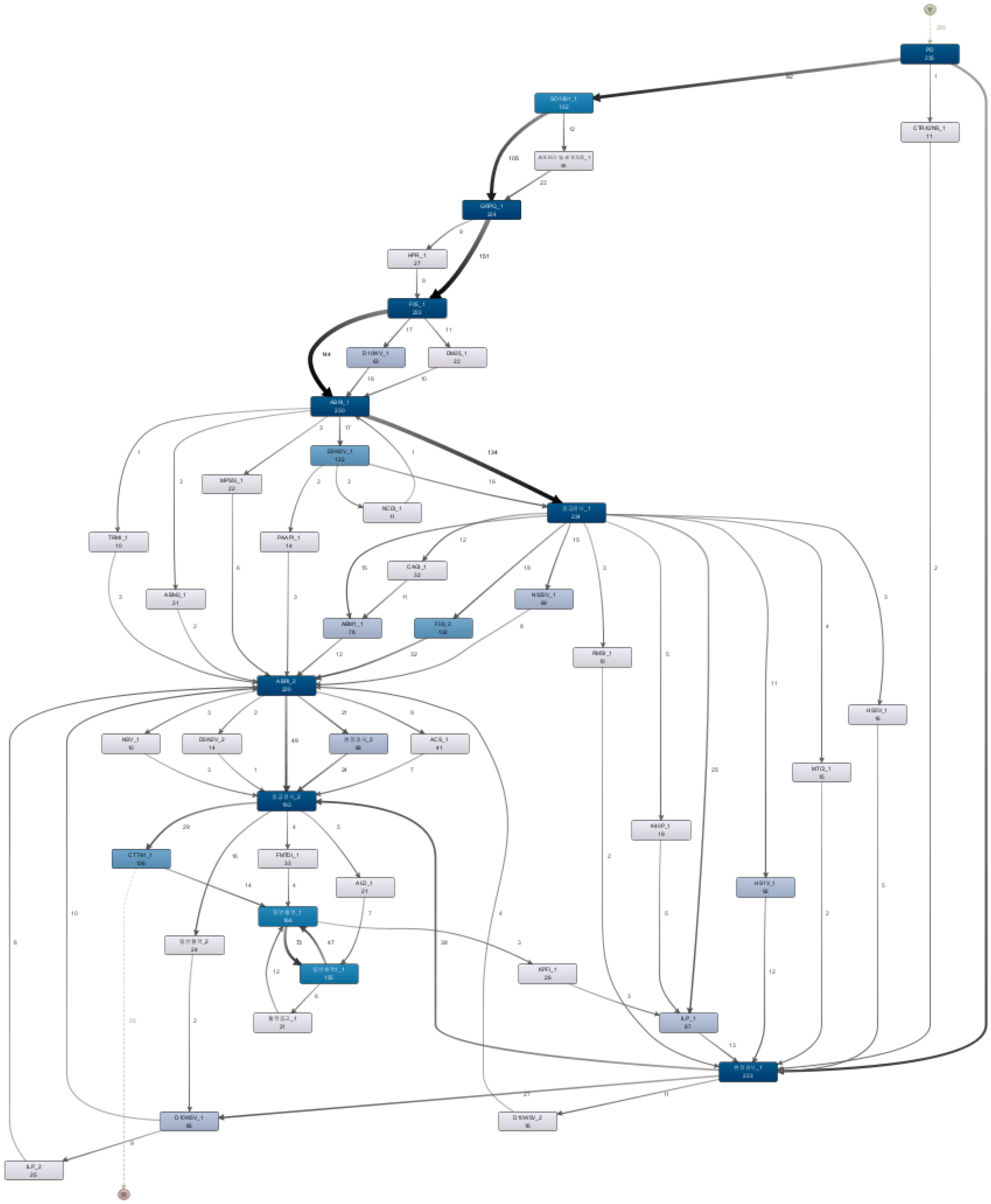

Supplement: S1 Fig — (PDF) [file pone.0279641.s002.pdf]
